# Preimplantation or gestation/lactation high-fat diet alters adult offspring metabolism and neurogenesis

**DOI:** 10.1093/braincomms/fcad093

**Published:** 2023-03-29

**Authors:** Diego A Ojeda, Oliver Hutton, Robert Hopkins, Felino Cagampang, Neil R Smyth, Tom P Fleming, Judith Eckert, Sandrine Willaime-Morawek

**Affiliations:** Faculty of Medicine, University of Southampton, Southampton General Hospital, Southampton SO16 6YD, UK; Faculty of Medicine, University of Southampton, Southampton General Hospital, Southampton SO16 6YD, UK; School of Biological Sciences, University of Southampton, Southampton General Hospital, Southampton SO16 6YD, UK; Faculty of Medicine, University of Southampton, Southampton General Hospital, Southampton SO16 6YD, UK; School of Biological Sciences, University of Southampton, Southampton General Hospital, Southampton SO16 6YD, UK; School of Biological Sciences, University of Southampton, Southampton General Hospital, Southampton SO16 6YD, UK; Faculty of Medicine, University of Southampton, Southampton General Hospital, Southampton SO16 6YD, UK; Faculty of Medicine, University of Southampton, Southampton General Hospital, Southampton SO16 6YD, UK

**Keywords:** nutrition, peri-conception, metabolic health, offspring long-term health, brain development

## Abstract

Poor maternal nutrition during pregnancy is known to impair fetal development. Moreover, the preimplantation period is vulnerable to adverse programming of disease. Here, we investigated the effect of a mouse maternal high-fat diet in healthy non-obese dams during preimplantation or throughout pregnancy and lactation on metabolism-related parameters and hippocampal neurogenesis in adult offspring. Female mice were fed from conception either a normal fat diet (normal fat diet group) or high-fat diet throughout gestation and lactation (high-fat diet group), or high-fat diet only during preimplantation (embryonic high-fat diet group, high-fat diet up to E3.5, normal fat diet thereafter). Maternal high-fat diet caused changes in the offspring, including increased systolic blood pressure, diurnal activity, respiratory quotient, and energy expenditure in high-fat diet females, and increased systolic blood pressure and respiratory quotient but decreased energy expenditure in high-fat diet males. High-fat diet males had a higher density of newborn neurons and a lower density of mature neurons in the dentate gyrus, indicating that exposure to a maternal high-fat diet may regulate adult neurogenesis. A maternal high-fat diet also increased the density of astrocytes and microglia in the hippocampus of high-fat diet males and females. Generally, a graded response (normal fat diet < embryonic high-fat < high-fat diet) was observed, with only 3 days of high-fat diet exposure altering offspring energy metabolism and hippocampal cell density. Thus, early maternal exposure to a fatty diet, well before neural differentiation begins and independently of maternal obesity, is sufficient to perturb offspring energy metabolism and brain physiology with lifetime consequences.

## Teaser

Ojeda *et al*. report that a maternal high-fat diet during the preimplantation period or throughout pregnancy and lactation, without obesity, leads to changes in metabolic-related parameters such as blood pressure, activity, respiratory quotient, and energy expenditure, and also in hippocampal neurogenesis and glial density in the adult offspring mice.

## Graphical Abstract

**Graphical abstract:**
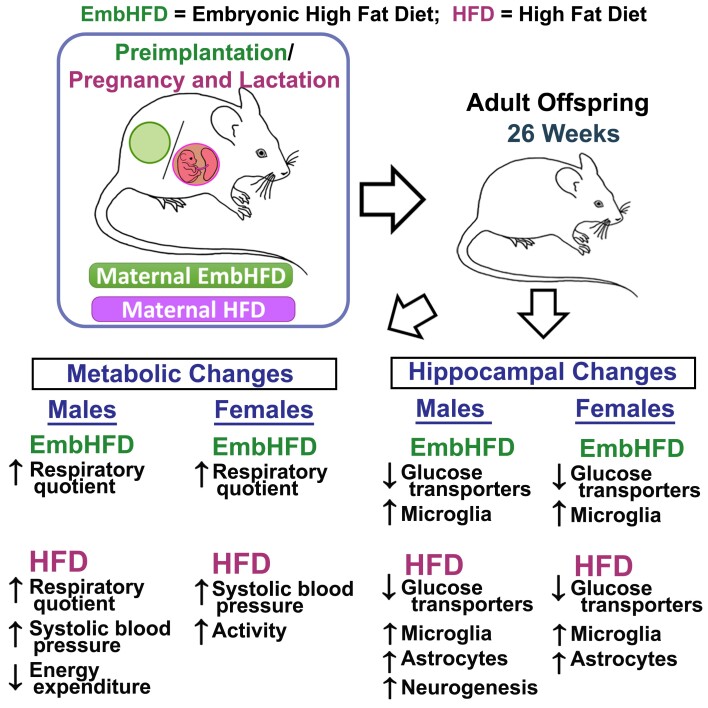

## Introduction

In recent years, the incidence of non-communicable diseases (NCDs), such as cardiovascular diseases, diabetes, obesity, some types of cancer, and neurological disorders, has increased considerably.^1,2^ It has been suggested that environmental factors are involved in increasing NCDs. Epidemiological and experimental research has indicated that early life and in-utero environments play an essential role in the origin of these diseases. The Developmental Origins of Health and Disease (DOHaD) hypothesis has been proposed to understand the implications of the maternal environment during pregnancy and offspring health.^3,4^ Also, the ‘predictive adaptive response’ hypothesis states that a mismatch between the prenatal and postnatal environment may be an important determinant leading to later metabolic diseases.^5^

The preimplantation period comprises the transition from fertilization through to the blastocyst before embryo implantation in the uterus. Observations from several animal models have suggested that changes in maternal diet or environment during the preimplantation period affect the pluripotent embryo and are sufficient to alter developmental potential leading to NCDs in later life mediated through epigenetic, metabolic and cellular processes.^6–9^ Notably, we showed previously that maternal undernutrition during preimplantation alone was sufficient to impair fetal brain development and cause adult memory deficit.^10^ There is mounting evidence that maternal obesity and consumption of a high-fat diet (HFD) during preimplantation and early pregnancy may increase the risk of developing metabolic diseases, and negatively affect brain function in offspring.^8,9,11–15^

Memory and learning are regulated in the central nervous system by different regions in the brain, with the hippocampus playing a predominant role in performing these functions. Additionally, neurogenesis occurs in the hippocampus, with newborn neurons replacing dying cells and forming functional synapses.^16,17^ The integrity of the hippocampus is critical to regulating processes such as learning and memory, anxiety, and spatial navigation. A nutritional imbalance is suggested to cause a negative effect on the hippocampus and, thus, on the number of proliferating cells and newborn neurons. Experimental evidence showed that HFD feeding could reduce neurogenesis in the hippocampus, just as maternal obesity can affect hippocampal neurogenesis in the offspring.^18–21^ However, the impact of a maternal HFD in the absence of obesity during preimplantation and pregnancy and lactation has not been extensively explored.

In this study, we hypothesized that maternal HFD altered neurogenesis and cellular organization in the hippocampus linked to changes in energy metabolism and offspring behaviour. Our study was performed in a non-obese model to assess whether maternal HFD alone, either during the preimplantation period (EmbHFD) or throughout pregnancy and lactation (HFD), was sufficient to adversely impact metabolism and brain function in offspring.

## Materials and methods

### Animal maintenance and diets

MF1 outbred mice were used to maintain a maximal level of heterozygosity, similar to what is seen in humans.^22^ Mice were bred in-house (University of Southampton, Biomedical Research Facility) and housed in standard cages with disposable bedding on a standard 12-hour light-dark cycle at constant temperature (22 ± 2°C) with food and water available *ad libitum*. All mice were fed with a standard lab chow diet. Non-obese virgin dams at 7.5–8.5 weeks old (28.3–45.4 grams) were left to mate overnight, and in the morning, a vaginal plug confirmed sexual intercourse (day E0.5). Mice and experimental procedures were conducted using protocols approved by, and in accordance with, the UK Home Office Animal (Scientific Procedures) Act 1986 and the local ethics committee at the University of Southampton, UK. The cohort was established for analysing the effects of a maternal HFD, and we present here some data related to metabolism and brain physiology from a subgroup of the cohort.

**Figure 1:**
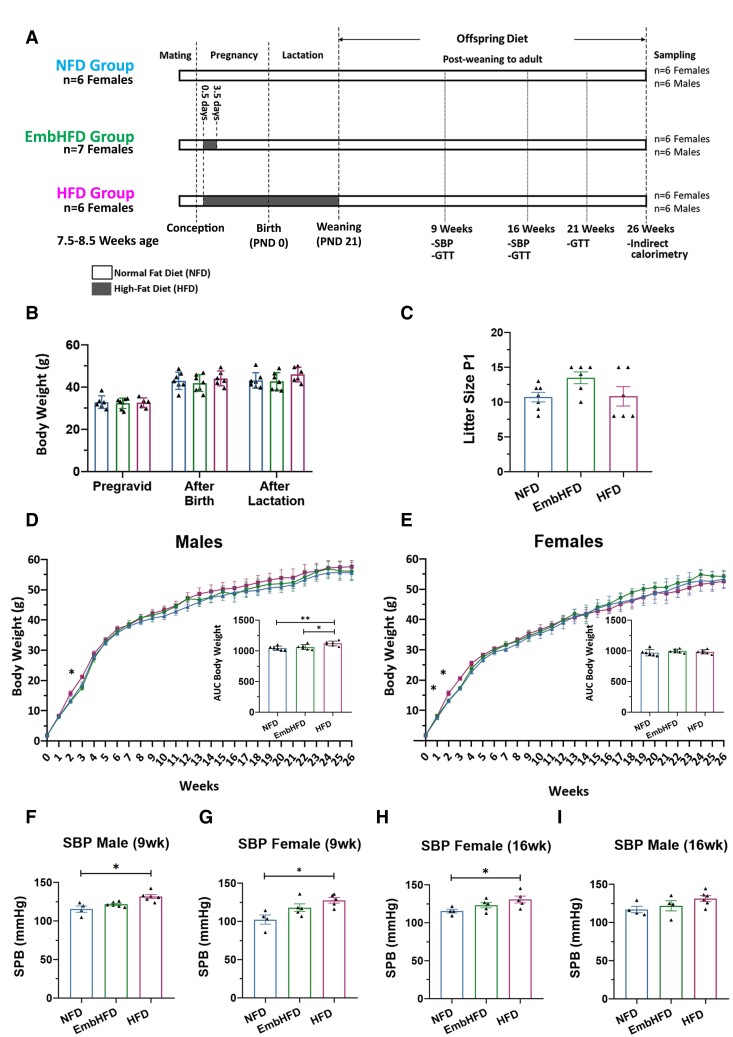
**Maternal HFD increases offspring SBP in the absence of obesity. A.** Schematic showing experimental design and feeding regimes for both dams and offspring. Female mice were fed either a control diet (NFD *n* = 6) or a HFD, during the preimplantation period (EmbHFD *n* = 7) or, pregnancy and lactation (HFD *n* = 6). Offspring were weaned at postnatal day 21. After weaning, all offspring were fed an NFD until their euthanasia at 26 weeks of age. **B.** No significant differences in maternal body weight were observed between the diet groups. NFD, *n* = 7; EmbHFD, *n* = 7; HFD, *n* = 6. Group comparisons via one-way ANOVA with Tukey’s multiple comparison test. **C.** No differences in offspring litter size at birth were observed. Each point represents the litter size per mother. NFD, *n* = 7; EmbHFD, *n* = 7; HFD, *n* = 6. Group comparisons via one-way ANOVA with Tukey’s multiple comparison test. **D.** Male offspring body weight over the 26 weeks of life. HFD males showed a higher BW at week 3 compared to NFD males (*P* = 0.024), NFD (blue triangles; *n* = 6) and EmbHFD (green dots; *n* = 6) HFD (red squares; *n* = 6). The data were analysed with two-way repeated-measures ANOVA followed by Tukey comparison. The variation of BW throughout the offspring’s life (AUC) showed that there are significant differences between NFD and EmbHFD males (*P* = 0.0087) and between the HFD and EmbHFD males (*P* = 0.0405). **E**. Female offspring body weight over the 26 weeks of life. HFD females showed a higher BW at weeks 2 and 3 compared to NFD females (*P* = 0.018 and *P* = 0.001 respectively). NFD (blue triangles; *n* = 6), EmbHFD (green dots; *n* = 6) HFD (red squares; *n* = 6). The data were analysed with two-way repeated-measures ANOVA followed by Tukey comparison. The variation of BW throughout the offspring’s life did not show significant differences between the groups. **F.** HFD males showed higher SBP than the NFD males (*P* = 0.0108) at 9 weeks of age, NFD, *n* = 4; EmbHFD, *n* = 6; HFD, *n* = 6. **G.** HFD females showed higher SBP than the NFD females (*P* = 0.0160) at 9 weeks of age, NFD, *n* = 4; EmbHFD, *n* = 5; HFD, *n* = 5. **H**. No significant differences were found in SBP in males at 16 weeks of age, NFD, *n* = 4; EmbHFD, *n* = 4; HFD, *n* = 6. **I.** HFD females had higher SBP than NFD females (*P* = 0.0407) at 16 weeks of age, NFD, *n* = 4; EmbHFD, *n* = 5; HFD, *n* = 5. Single data points for each mouse were represented as black dots, (f, g, h and i), and data were analysed by multilevel random effects regression. NFD group in blue, the EmbHFD group in green and HFD group in red. Data are expressed as mean ± SEM. *P < 0.05, **P < 0.01.

After pregnancy confirmation, 6–7 dams were randomly assigned to each diet group using a computer-based random order generator. Control dams were fed a normal fat diet (NFD group, *n* = 7); rat and mouse no.1 maintenance diet, obtained from Special Diet Services, Essex, UK (SDS) containing 7.5%Kcal fat, 17.5%Kcal protein, 75%Kcal carbohydrates. Experimental dams were fed HFD (SDS diet code:824053) containing 45%Kcal fat, 20%Kcal protein and 35%Kcal carbohydrate (for detailed diet compositions, see Supplementary Table 1). The HFD was maintained either throughout gestation and lactation (HFD group, *n* = 6) or switched at E3.5, corresponding to the blastocyst stage, to NFD (EmbHFD group, *n* = 7). Litters were normalised to three males and three females at birth and weaned at 3 weeks. Each litter of males and females were caged separately and fed NFD after weaning until 26 weeks of age. For analysis, one female and one male were randomly selected per litter to reach six offspring per dietary group per sex. The experimental design is illustrated in Fig. 1A. Offspring body weight was recorded weekly for 26 weeks. The index of obesity degree [IOD = (body weight of mouse fed with HFD—average body weight of mice in the control group)/average body weight of mice in the control group × 100%] was used to identify obese mice at 26 weeks (IOD > 20%).^23^ Systolic blood pressure (SBP) was measured at postnatal weeks 9 and 16 by tail-cuff plethysmography with a non-invasive blood pressure monitor (NIBP-8, Columbus Instruments, USA) in a pre-warmed room (28–30°C), and mice were acclimatized for 90 min.^24^ Five SBP readings with good waveforms and good overall quality were taken per mouse, and the mean value of the three middle readings was calculated and recorded. Glucose tolerance tests (GTT) were conducted at postnatal weeks 9, 16 and 21 in unrestrained conscious mice after a 12-hour overnight fast, with access to water. Glucose was measured in small blood drops collected by tail tipping using a blood glucose metre (Accu-Chek Aviva, Roche Diagnostics GmbH, Germany). Topical anaesthetic cream (Lidocaine 5%, Teva, UK) was applied to the tail 20 min before starting the GTT. After the recording baseline (fasting) glucose level (0 min), a glucose (G8270, Sigma, UK) solution (20%, in sterile distilled water) was injected intraperitoneally at a dose of 2 g/kg. Blood glucose levels were measured at 15, 30, 60 and 120 min after glucose administration. Area under the curve (AUC) values were calculated by the trapezoidal rule.^24^

### Indirect calorimetry

Indirect calorimetry was measured at 26 weeks using a closed modular indirect calorimetric system (Oxylet, Panlab, Spain). Each mouse was placed in an individual metabolic chamber with free access to food and water. Oxygen consumption (vO_2_) and carbon dioxide production (vCO_2_) were recorded at 5 min intervals using the Oxylet-LE 405-gas analyser and Metabolism software 2.1.02 computer-assisted data acquisition program (Oxylet, Panlab, Spain) over two 24-h periods. Energy expenditure (EE) was calculated as follows: EE (kcal/day/kg^0.75^) = vO_2×_1.44×[3.815 + (1.232×RQ)]. The respiratory quotient (RQ) was calculated by dividing the vCO_2_ produced by the vO_2_ consumed (RQ = vCO_2_/vO_2_). A transducer was located below the cage to detect all activity variations (Oxylet-LE 1335-Physiocage, PANLAB). The system calculated the activity of each animal by detecting changes in potential and kinetic energy in its movements.^25,26^

### Tissue collection

At 26 weeks, between 09.00 and 11.00 am, mice were trans-cardially perfused with 0.9%NaCl containing 5 U/mL heparin sodium (CP Pharmaceuticals) and their brains and livers were removed. Livers were weighed, snap-frozen in liquid nitrogen and stored at −80°C. Brains were cut sagitally, with one hemisphere used to dissect and snap-freeze the hippocampus in liquid nitrogen and the other half fixed in 4% paraformaldehyde for 12–24 hours and then stored in 30% sucrose. Fixed hippocampus samples were then embedded in optimum cutting temperature (Tissue Tek) and stored at −80°C until sectioning. Coronal brain sections (14 µm thick) between bregma −1.22 mm and bregma −2.70 mm were cut on a Leica cryostat and stored onto Superfrost Plus slides (Fisher Scientific) at −20°C.

### Immunohistochemistry and image analysis

Immunohistochemistry was performed as previously published.^10^ Briefly, sections were treated with 10% donkey serum and 0.5% bovine serum albumin in phosphate-buffered saline for non-specific binding. After rinses with phosphate-buffered saline -0.1% TritonX-100 (PBST), sections were incubated overnight at 4°C with the following antibodies: rabbit anti-NeuN (Merck Millipore, MAB377, 1:200), rabbit anti-GFAP (Dako, Z0334, 1:500), rabbit anti-Iba1 (Wako, 019–19741, 1:500), goat anti-doublecortin (DCX) (Santa Cruz, Sc-8066, 1:100), or goat anti-Sox2 (Santa Cruz, Sc-17320, 1:100). Sections were incubated at 37°C for 2 h with the appropriated conjugated secondary antibody: AlexaFluor 488− or 568− donkey anti-mouse, anti-rabbit or anti-goat IgG. Nuclei were counterstained with 4,6-diamidino-2-phenylindole (DAPI) for 5 min before mounting the slides. Mowiol^®^4–88 Reagent (Calbiochem, Darmstadt, Germany) was used as the mounting medium. Sections were imaged with a Leica DM5000B microscope coupled to a Leica DFC300FX camera. For each marker, three sections per animal were analysed using ImageJ. Cell densities were analysed by counting positive cells in different regions of interest. Four regions of interests were delineated to cover the different parts of the hippocampus: Cornu ammonis 1 (CA1), Cornu ammonis 3 (CA3), dentate gyrus (DG) [subgranular zone (SGZ) and granule cell layer (GCL)] and hilus. All analysis was performed blinded to the diet group.

### Quantitative real-time PCR

Total RNA was isolated from frozen hippocampal and liver samples using an RNeasy Lipid Tissue Mini kit (Qiagen, UK) according to the manufacturer's instructions.^10^ The isolated RNA was quantified using a Nanodrop ND-1000 spectrophotometer (LabTech UK), and only samples with an adequate RNA concentration (A260/A280⩾1.8) and purity (A230/A260⩾2.0) were selected for reverse transcription. 300 ng RNA from each sample was reverse transcribed using a PrecisionReverse-Transcription^®^ Premix kit (Primer Design, UK) following the manufacturer's protocol. cDNA was stored at −20°C. cDNA libraries were analysed by qPCR on a CFX96 Real-Time System (Bio-Rad, Hercules, CA) using the Precision^®^ Plus-SYBRgreen (PrimerDesign, UK), gene-specific primers (0.5 µM each), and 10 ng cDNA. Melting curve analysis was carried out to verify the amplification specificity. The reference genes (hippocampus: *Fbxw2, Pak1lp1*, *Ap3d1* and liver: *Pgk1, Tbp*) were selected using a 12 geNormPlus kit (PrimerDesign, UK). Finally, data analysis was performed using the comparative CT method (2^−ΔΔCT^), and assays were performed in triplicate to control for technical errors. Gene expression of different markers was quantified using specific primers as listed in Supplementary Table 2 (Sigma Aldrich, UK), or primers from PrimerDesign: *Ap3d1, B3-Tub*, *Fbxw2, Notch-1, Pak1lp1, Pax6*, *Pgk1* and *Tbp* (sequences are property of PrimerDesign, UK). All primers were designed according to the MIQE guidelines. To assess mRNA expression of reference genes, the qbase + software package (BioGazelle, Belgium) was used. The expression stability value of gene expression (*M*-value lower than 0.5) and the variation in stability of reference genes (*V*-value lower than 0.2) were analysed to evaluate the lowest number of genes required for accurate normalization.

### Western blot analysis

Hippocampal samples were homogenized in radioimmunoprecipitation assay buffer (Thermo Fisher Scientific, USA) with protease inhibitors (EASYpack, Roche, Germany) and phosphatase inhibitors (PhosSTOP, Roche, Germany). Homogenates were centrifuged at 1000 g, and the supernatant was collected. Proteins were quantified using BCA assay (Thermo Fisher, USA) following the manufacturer's instructions. Western blots were performed on 20μg total protein lysates loaded on 10–20% Criterion TGX Stain-Free Precast gels (Bio-Rad, CA, USA) and transferred to a 0.45-μm PVDF membrane (Bio-Rad, CA, USA). The membranes were blocked with 5% fat-free milk in PBST for one hour at 4°C and incubated overnight with anti-GFAP (rabbit, 1:10 000; Dako), and anti-GAPDH (mouse, 1:10 000; abcam) at 4°C. The membranes were incubated for 1 h with secondary antibodies IRDye^®^680RD Goat anti-mouse and IRDye^®^800CW Goat anti-rabbit. The detection of the bands was performed using the Odyssey imaging system (LI-COR). Densitometric quantification of protein bands was performed with Image Studio Lite, version 5.2 (Li-Cor, Lincoln, NE, USA).

### Statistical analysis

All experiments and analyses were performed blinded to the diet group. Dams body weight and litter size data were compared using an ordinary one-way ANOVA followed by a Tukey–Kramer multiple comparisons test. Offspring data were compared between groups using a multilevel random effects regression model accounting for different parameters (litter size, sex and body weight) from individual animals (SPSS version 26). Thus, differences identified between treatment groups throughout the study are independent of maternal origin, litter size, and body weight.^24,27,28^ Correlation between the number of microglia cells and DCX–/NeuN+ cells was calculated using the Pearson correlation coefficient. The normality of data was tested using the Shapiro–Wilk test. All data are expressed as the mean ± standard error of the mean (SEM). All tests were performed using a significance level of α = 0.05 with 95% confidence to determine significant group mean differences. A power analysis based on previously published data was performed to estimate the sample size.^29,30^*P-*value is shown as *P < 0.05, **P < 0.01, ***P < 0.001, ****P ≤ 0.0001.

## Results

### Maternal HFD had minimal effect on offspring body weight but increased adult offspring systolic blood pressure

Maternal body weight was measured at three-timepoints (pregravid, after birth and at the end of lactation). No significant changes in maternal body weight were observed (Fig. 1B). Litter size at birth was not different between the diet groups (Fig. 1C). Offspring body weight was measured weekly during the entire experiment and, body weights were only significant at 3 weeks in HFD males (*P* = 0.024) and 2- and 3 weeks in HFD females (*P* = 0.018 and *P* = 0.001, respectively) compared to NFD males and females respectively. However, HFD males had an increased body weight AUC compared to EmbHFD (*P* < 0.05) and NFD (*P* < 0.01) males (Fig. 1D and E). Despite the small body weight increase in HFD male offspring, the mice were not considered overweight or obese at 26 weeks (NFD: 55.6 ± 2.24 g, EmbHFD: 56.1 ± 3.02 g, HFD: 57.6 ± 2.08 g, as mean ± SEM). Also, the IOD did not indicate obesity at 26 weeks (EmbHFD males 0.92%, HFD males 3.64%, EmbHFD females 1.60%, HFD females 1.35%), confirming that the animals did not develop obesity.

No changes were observed in offspring for blood glucose levels in the GTT at postnatal weeks 9, 16 and 21 (Supplementary Fig. 1). SBP was higher in HFD females than NFD females at 9 and 16 weeks (*P* < 0.05) and in HFD males than NFD males at 9 weeks (*P* < 0.05) (Fig. 1F–I). No difference in SBP was found in EmbHFD males or females at 9 and 16 weeks although consistently higher than NFD. These data indicate that our model did not induce maternal or offspring obesity, but maternal HFD led to a selected increases in offspring weight and SBP suggesting effects on aspects of offspring metabolism.

### Maternal HFD increased diurnal locomotor activity in female adult offspring

**Figure 2:**
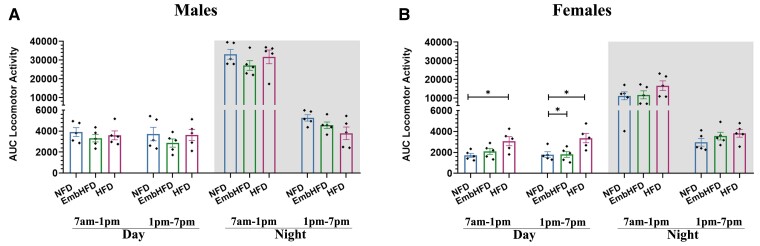
**Effect of maternal HFD and EmbHFD on locomotor activity in the adult offspring. A.** Male locomotor activity over a 24 h period (12 h light and 12 h dark). The bars represent the AUC of the activity during the day and night cycle, over a 24 h period. NFD, *n* = 5; EmbHFD, *n* = 5; HFD, *n* = 5. Data were analysed by multilevel random effects regression **B.** Female locomotor activity over a 24 h period (12 h light and 12 h dark). HFD females had higher activity during the day compared with the NFD females. The HFD and EmbHFD females were different in the second part of the day. The bars represent the AUC of the activity during the day and night cycle, over a 24 h period. NFD, *n* = 5; EmbHFD, *n* = 5; HFD, *n* = 5. Data were analysed by multilevel random effects regression. The shaded areas demarcate the dark phases (from 7:00 pm to 7:00 am). NFD group (blue), EmbHFD group (green) and HFD group (red) at 26 weeks of age. Single data points for each mouse are represented as black dots, and bars represent the AUC for each diet group. All points represent means ± SEM. Each animal belonged to a single litter. *P < 0.05.

We evaluated the effect of the maternal diet on the offspring spontaneous activity. No differences were observed between the male groups (Fig. 2A, Supplementary Fig. 2A). HFD females had higher diurnal activity (7:00 am–1:00 pm *P* = 0.0174, 1:00 pm–7:00 pm *P* = 0.0266) compared with NFD females (Fig. 2B, Supplementary Fig. 2B). No differences were found for EmbHFD females during the day, and for HFD or EmbHFD females during the night, compared to NFD females. At night, males in all diet groups had higher activity values than females (Supplementary Table 3).

### Maternal HFD and EmbHFD impair respiratory quotient and energy balance in the adult offspring

**Figure 3:**
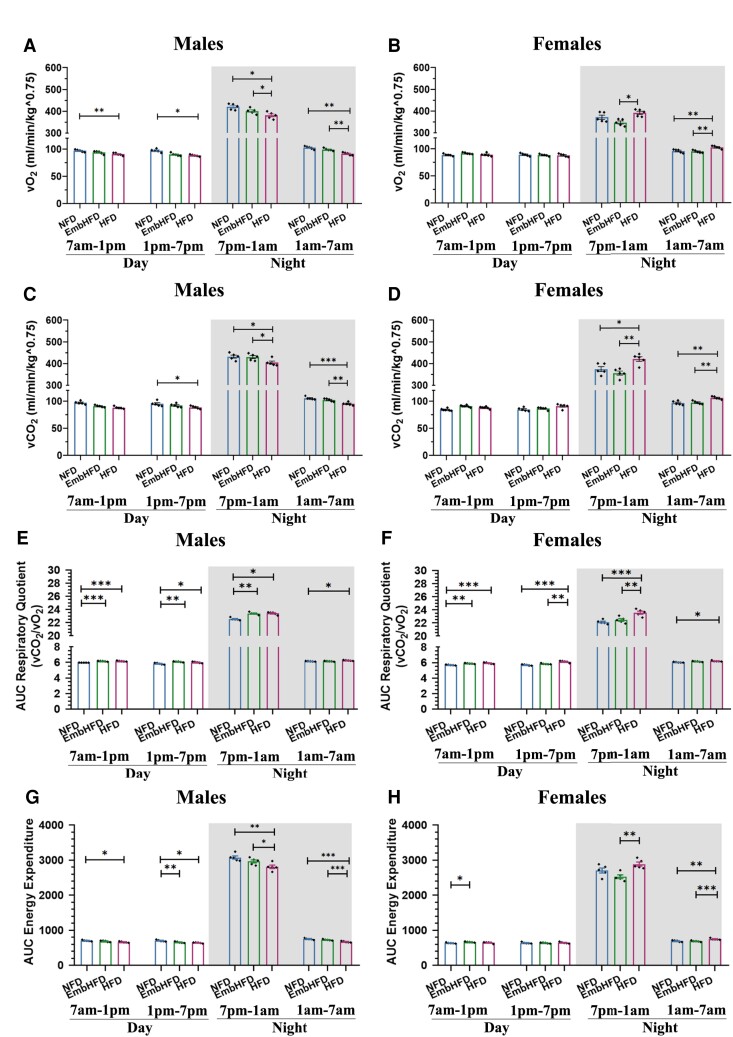
**Effect of maternal HFD and EmbHFD on indirect calorimetry in the adult offspring. A.** Male vO_2_ consumption over day/night-time cycle. In males, the HFD group showed a smaller VO_2_ AUC during the first and second part of the day, and the first and second part of the night when compared to the NFD group, and EmbHFD males had a decreased VO_2_ AUC in the second part of the day when compared with the NFD group. The bars represent the AUC of the oxygen consumption during the day and night cycle, over a 24 h period. NFD, *n* = 5; EmbHFD, *n* = 5; HFD, *n* = 5. Data were analysed by multilevel random effects regression. **B.** Female vO_2_ consumption over day/night-time cycle. In females, the HFD group showed a higher vO_2_ AUC in the second part of the night compared to the NFD group. The bars represent the AUC of the oxygen consumption during the day and night cycle, over a 24 h period. NFD, *n* = 5; EmbHFD, *n* = 5; HFD, *n* = 5. Data were analysed by multilevel random effects regression **C.** Male carbon dioxide vCO_2_ consumption over a 24 h period (12 h light and 12 h dark). In males, the vCO_2_ AUC was lower in the second part of the day, in the first and second part of the night in the HFD group compared to the NFD group. Also, there was a significant difference between EmbHFD and HFD males in the first part, and the second parts of the night. The bars represent the AUC of the carbon dioxide production during the day and night cycle, over a 24 h period. NFD, *n* = 5; EmbHFD, *n* = 5; HFD, *n* = 5. Data were analysed by multilevel random effects regression. **D.** Female vCO_2_ consumption over a 24 h period (12 h light and 12 h dark). In females, there was a significant difference between the HFD and the NFD group during the first and second part of the night. There is a significant difference between EmbHFD and HFD females in the first and the second part of the night. The bars represent the AUC of the carbon dioxide production during the day and night cycle, over a 24 h period. NFD, *n* = 5; EmbHFD, *n* = 5; HFD, *n* = 5. Data were analysed by multilevel random effects regression. **E.** Male RQ over day/night-time cycle. In males, an increase in RQ AUC values during the first and second part of the day compared to the NFD group. Similarly, an increased RQ AUC was observed in the first part and second part of the night when compared to the NFD group. EmbHFD mice had a significantly higher RQ during the first and second parts of the day compared to the NFD group. The bars represent the AUC of the RQ during the day and night cycle, over a 24 h period. NFD, *n* = 5; EmbHFD, *n* = 5; HFD, *n* = 5. Data were analysed by multilevel random effects regression. **F.** Female RQ over day/night-time cycle. In females, there is an increase in RQ AUC during the first and second parts of the day, and in the first and second parts of the night when compared to the NFD group. EmbHFD mice also showed a greater RQ AUC in the first part of the day compared to the NFD group. HFD females and EmbHFD females were significantly different in the second part of the day, and the first part of the night (*P* = 0.007). The bars represent the AUC of the RQ during the day and night cycle, over a 24 h period. NFD, *n* = 5; EmbHFD, *n* = 5; HFD, *n* = 5. Data were analysed by multilevel random effects regression. **G.** Male EE over a day/night-time cycle. In males, the HFD group showed a lower EE AUC during the first and second part of the day, and the first and second part of the night, whereas, in the EmbHFD group, the EE AUC was only reduced in the second part of the day in comparison with the NFD group. It was a significant difference between EmbHFD and HFD males in the first part, and the second part of the night. The bars represent the AUC of the EE during the day and night cycle, over a 24 h period. NFD, *n* = 5; EmbHFD, *n* = 5; HFD, *n* = 5. Data were analysed by multilevel random effects regression. **H.** Female EE over day/night-time cycle. In females, the EmbHFD group has a significant EE increase in the first part of the day, with respect to the NFD group, and during the night period, the HFD females showed an increase only in the second part of the night. There is a significant difference between EmbHFD and HFD females in the first part, and the second part of the night. The bars represent the AUC of the EE during the day and night cycle, over a 24 h period. NFD, *n* = 5; EmbHFD, *n* = 5; HFD, *n* = 5. Data were analysed by multilevel random effects regression. The shaded areas demarcate the dark phases (from 7:00 pm to 7:00 am). NFD group (blue), EmbHFD group (green) and HFD group (red) at 26 weeks of age. Single data points for each mouse are represented as black dots, and bars represent the AUC for each diet group. All points represent means ± SEM. Each animal belonged to a single litter. *P < 0.05, **P < 0.01, ***P < 0.001, ****P ≤ 0.0001.

As changes in activity might be reflected by changes in O_2_ consumption and CO_2_ production, these were also analysed. HFD males consumed less oxygen during the day (first part *P* = 0.0043, second part *P* = 0.0005) and night (first part *P* = 0.0010, second part *P* = 0.0001), while EmbHFD males consumed less oxygen in the second part of the day (*P* = 0.0012), compared to NFD males. There is a significant difference between EmbHFD and HFD males in the first part (*P* = 0.039), and the second part (*P* = 0.002) of the night (Fig. 3A, Supplementary Fig. 2C). HFD females consumed more O_2_ in the second part of the night (*P* = 0.0089) compared to the NFD females (Fig. 3B, Supplementary Fig. 2D) and during the whole night compared to EmbHFD females (first part *P* = 0.008, second part *P* = 0.004). Also, HFD males produced less CO_2_ compared to NFD males (second part day, *P* = 0.0446), and the whole night (first part *P* = 0.0259, and second part *P* = 0.0002) (Fig. 3C, Supplementary Fig. 2E). HFD females produced more CO_2_ at night (first part *P* = 0.0306, and second part *P* = 0.0042) compared to both NFD and EmbHFD females (first part *P* = 0.004, and second part *P* = 0.001) (Fig. 3D, Supplementary Fig. 2F). Sex differences were observed with females consuming less O_2_ and producing less CO_2_ than males (Supplementary Table 3).

RQ corresponds to the vCO_2_ produced, divided by the vO_2_ that was consumed simultaneously (RQ = VCO_2_/VO_2_). As expected, all the animals demonstrated greater RQ values at night compared to the day, since the feeding and activity pattern of these animals is nocturnal (Fig. 3E and F, Supplementary Fig. 2G and H). HFD males (day: first *P* = 0.0001, second part *P* = 0.0327; night: first *P* = 0.0001; second part *P* = 0.0062) and females (day: first *P* = 0.0009, second part *P* = 0.0003; night: first part *P* = 0.0015) had a higher RQ compared to NFD groups across the 24-hour cycle. EmbHFD males had a higher RQ during the day (first *P* = 0.0001, second part *P* = 0.0032) and the first part of the night (*P* = 0.0003) compared to NFD males (Fig. 3E, Supplementary Fig. 2G). EmbHFD females had a higher RQ during the first part of the day (*P* = 0.0013) compared to NFD females (Fig. 3F, Supplementary Fig. 2H). In terms of sex differences, EmbHFD males had higher RQ than EmbHFD females in the first part of the night (Supplementary Table 3).

EE was assessed by indirect calorimetry, quantifying vO_2_ consumption and vCO_2_ production over the day/night cycle. HFD males had lower EE during the day (first *P* = 0.0109, second part *P* = 0.0021) and night (first *P* = 0.0023, second part *P* = 0.0001), compared to NFD males. EmbHFD males had reduced EE in the first (*P* = 0.0066) and the second part of the day (*P* = 0.0099) compared to NFD males (Fig. 3G, Supplementary Fig. 2I). The EmbHFD females had increased EE compared to NFD females, in the first part of the day (*P* = 0.032). HFD females had increased EE compared to EmbHFD females during the night (first *P* = 0.006, second *P* = 0.0002 part) (Fig. 3H, Supplementary Fig. 2J). Regarding sex effect, NFD males had higher EE during day and night compared to NFD females.

Collectively, these calorimetric results suggest maternal HFD and EmbHFD treatments alter offspring adult female activity and both male and female EE in a treatment and sex-dependent manner. We next investigated dietary effects on offspring expression of key genes regulating energy metabolism.

### Maternal HFD altered gene expression related to energy metabolism in the offspring liver

**Figure 4:**
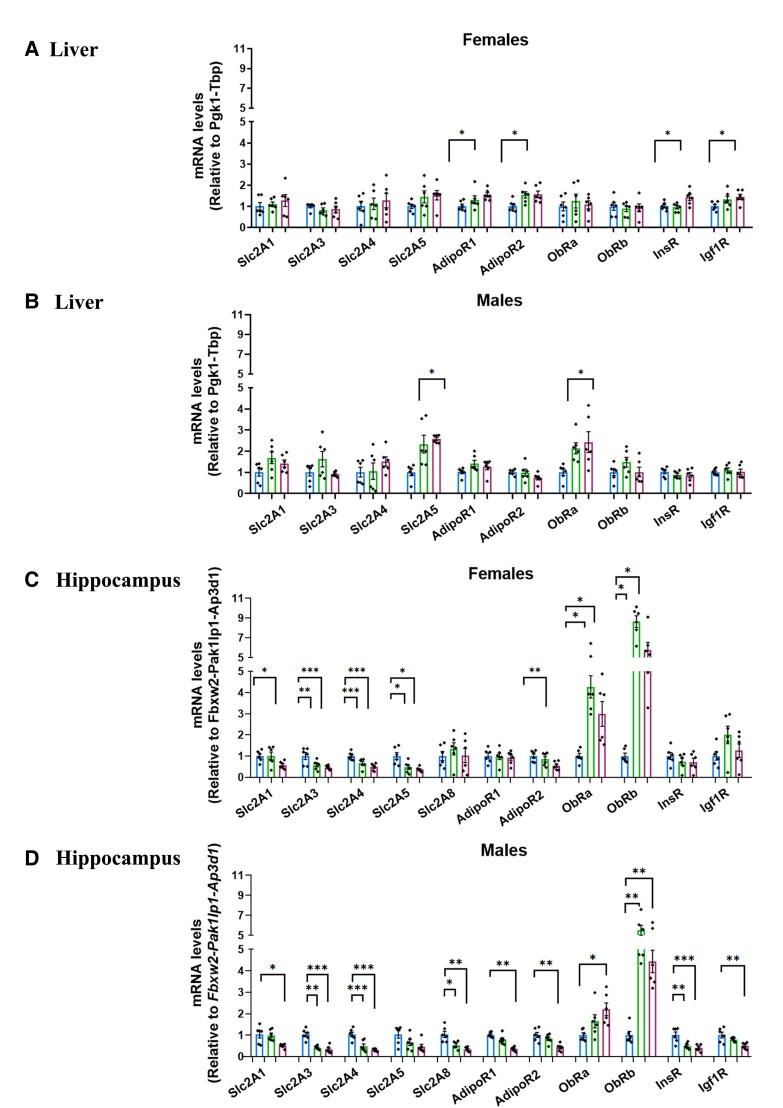
**Maternal HFD and EmbHFD affect the expression of metabolic markers in the adult liver and hippocampus. A.** In the liver, mRNA levels of *ObRa* were increased in the HFD males compared to the NFD males. **B.** In the liver, mRNA levels of *AdipoR1*, *AdipoR2*, *InsR* and *Igf1R* were increased in the HFD females compared to the females. **C.** In the hippocampus, mRNA levels of *Slc2A1, Slc2A3, Slc2A4, Slc2A8*, *AdipoR1*, *AdipoR2*, *InsR* and *Igf1R* were reduced, but the expression of *ObRa*, and *ObRb* was increased in the HFD males compared to the NFD males. EmbHFD males showed lower mRNA levels of *Slc2A3*, *Slc2A4*, *Slc2A8* and *InsR*, and higher expression levels of *ObRb* compared to the NFD males. **D.** In the hippocampus, HFD females showed lower mRNA levels than the NFD group of *Slc2A1*, *Slc2A3*, *Slc2A4*, *Slc2A5* and *AdipoR2* genes, and higher mRNA levels of *ObRa*, and *ObRb* genes. mRNA levels of *Slc2A3*, *Slc2A4* and *Slc2A5* genes in EmbHFD females were significantly reduced, but mRNA levels of *ObRa*, and *ObRb* were significantly increased compared to the NFD males. In the liver, the mRNA levels of the selected markers were measured by qPCR and normalized to *Pgk1* and *Tbp* and to *Fbxw2, Pak1lp1* and *Ap3d1* in the hippocampus. Data were analysed by multilevel random effects regression and shown as dot plots with mean and SEM (**A, B, C**, and **D**). NFD, *n* = 6; EmbHFD, *n* = 6; HFD, *n* = 6 (**A, B, C** and **D**). NFD group (blue), EmbHFD group (green) and HFD group (red) at 26 weeks of age. Each animal belonged to a single litter. *P < 0.05, **P < 0.01, ***P < 0.001.

The liver plays a critical role in regulating energy balance, and glucose and lipid metabolism.^31^ We analysed the effect of diets on offspring liver mRNA expression of glucose transporters encoded by the *SLC2* genes including *Slc2A1*, *Slc2A3*, *Slc2A4* and *Slc2A5*^32^ but no differences were found (Fig. 4A and B). Leptin is known to regulate energy homeostasis in the CNS and peripheral tissues such as the liver, and this is mediated via leptin receptors (ObRa and ObRb).^33,34^ In the liver, adiponectin controls glucose and lipid metabolism by stimulating glycolysis and fatty acid oxidation.^35^ Receptors for different hormones related to the control of energy metabolism also include adiponectin (*AdipoR1* and *AdipoR2*), insulin (*InsR*) and insulin-like growth factor 1 (*Igf1R*). Male HFD offspring showed increased *ObRa* mRNA (*P* = 0.032) (Fig. 4A), while female HFD offspring had increased *adipoR1* (*P* = 0.0368), *adipoR2* (*P* = 0.0492)*, InsR* (*P* = 0.0449), and *Igf1R* (*P* = 0.0236) (Fig. 4B). No differences were found in EmbHFD offspring. These results suggest that maternal HFD in the absence of obesity alters the expression of liver glucose transporters and metabolic regulators in a sex-specific manner.

### Maternal HFD and EmbHFD altered gene expression of glucose transporters, adiponectin, leptin and insulin receptors in hippocampus of offspring

Glucose is the primary energy source in the brain, and glucose receptors facilitate the transport and uptake of glucose into neurons and glial cells.^36^ In male HFD offspring, expression of *Slc2A1* (*P* = 0.006), *Slc2A3* (*P* = 0.0001), *Slc2A4* (*P* = 0.0001) and *Slc2A8* (*P* = 0.002) were all reduced in the hippocampus compared to NFD males, while EmbHFD male offspring showed lower expression of *Slc2A3* (*P* = 0.001), *Slc2A4* (*P* = 0.0002) and *Slc2A8* (*P* = 0.013) (Fig. 4C). HFD female offspring showed lower levels for *Slc2A1* (*P* = 0.039), *Slc2A3* (*P* = 0.002), *Slc2A4* (*P* = 0.0003) and *Slc2A5* (*P* = 0.0003) in the hippocampus while EmbHFD females expressed lower levels of *Slc2A3* (*P* = 0.006), *Slc2A4* (*P* = 0.010) and *Slc2A5* (*P* = 0.018) (Fig. 4D).

HFD males had reduced expression of *AdipoR1* (*P* = 0.0001), *AdipoR2* (*P* = 0.002), *InsR* (*P* = 0.001) and *Igf1R* (*P* = 0.004) and higher expression of *ObRa* (*P* = 0.013) and *ObRb* (*P* = 0.002) in the hippocampus, while EmbHFD male offspring showed lower mRNA levels of *InsR* (*P* = 0.006), and higher mRNA levels of *ObRb* (*P* = 0.0002) (Fig. 4C). EmbHFD female offspring exhibited increased mRNA levels of *ObRa* (*P* = 0.049), and *ObRb* (*P* = 0.008) while HFD females showed lower mRNA levels of *AdipoR2* (*P* = 0.017) and higher mRNA levels of *ObRa* (*P* = 0.023) and *ObRb* (*P* = 0.0002) (Fig. 4D). Collectively, maternal HFD and EmbHFD alter the expression of genes related to energy metabolism (glucose, adiponectin and leptin signalling markers) in the brain and/or liver. These results may indicate the association between adiposity/circulating lipid levels and maternal diet; therefore, a future analysis that allows quantifying serum lipid levels is necessary, as adiponectin plays a key role in insulin sensitivity, glucose utilization, and fatty acid oxidation.^35,37^

### Maternal HFD increased type-1 stem cell density in offspring hippocampus

**Figure 5:**
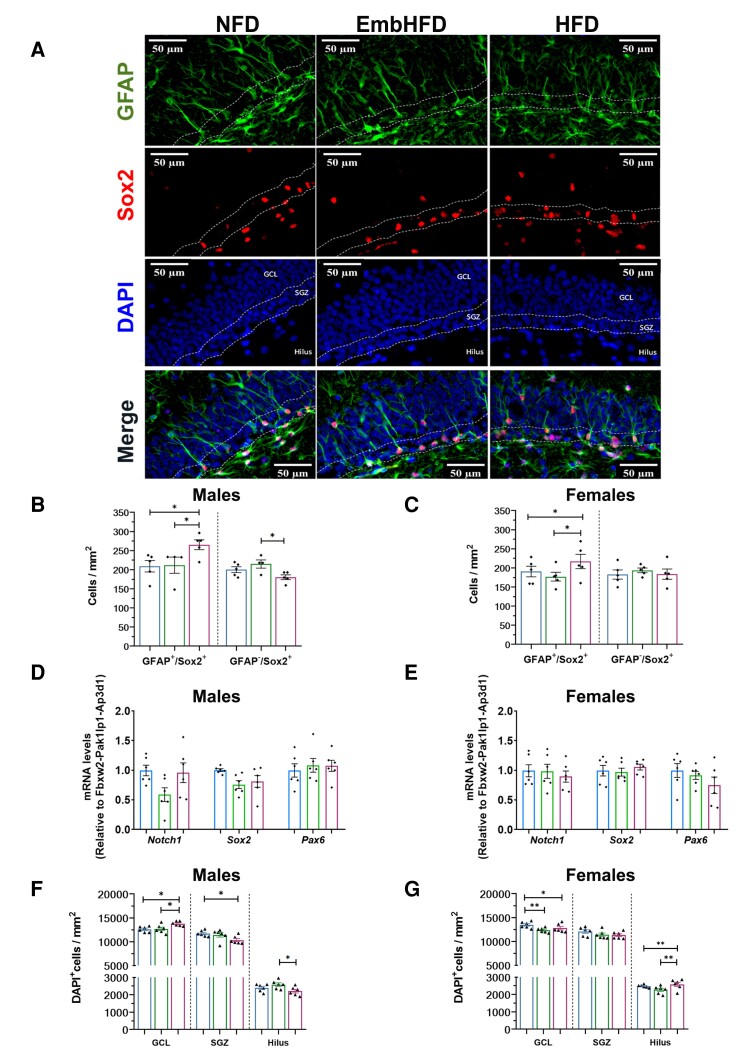
**Maternal HFD and EmbHFD increase type-1 stem cell density in the male and female adult offspring hippocampus. A.** Images of coronal SGZ sections analysed by GFAP (green top row), SOX2 (red second row), DAPI (blue third row), and merge (bottom row). **B.** In males, the HFD group had higher GFAP+/SOX2+ cell density in comparison to the NFD males. There is a significant difference between the EmbHFD males and the HFD males in the density of GFAP+/SOX2+, and GFAP-/SOX2+ cells. **C**. In females, the HFD group had higher GFAP+/SOX2+ cell density in comparison to the NFD females. There is a significant difference between the EmbHFD females and the HFD females in the density of GFAP+/SOX2+ cells. Hippocampal mRNA levels of *Notch1*, *Sox2* and *Pax6* were assessed, but no differences were observed in males in **D** or females in **E**. **F.** Total cell density quantified by DAPI staining showed in the HFD males an increase in the GCL and decrease in the SGZ compared to the NFD group, and there is a significant difference between the EmbHFD males and the HFD groups in the GCL and hilus. **G.** HFD females exhibited lower cell density in the GCL, and higher cell density in the Hilus compared to the NFD group, there was also a decrease in the cell density in the GCL in the EmbHFD females, and EmbHFD females and the HFD females in the hilus. NFD group (blue), EmbHFD group (green) and HFD group (red). Data were analysed by multilevel random effects regression and shown as dot plots with mean and SEM (**A, B, C** and **D**). NFD, *n* = 6; EmbHFD, *n* = 6; HFD, *n* = 6 (**B, C, D, F** and **G**). Each animal belonged to a single litter. GCL; granule cell layer; SGZ; subgranular zone Scale bars, 50 μm. The mRNA levels of the selected markers were analysed by qPCR and normalized to *Fbxw2, Pak1lp1* and *Ap3d1*. *P < 0.05, **P < 0.01, ***P < 0.001.

As *Slc2A8, AdipoR1* and *AdipoR2* genes have been shown to regulate hippocampal neurogenesis^38–40^ and their expression was decreased in HFD males, we analysed neural stem cells and neurogenesis in the hippocampus. Type-1 and type-2 stem cell density analysis in the DG SGZ were based on their localization and cell morphology (Fig. 5A).^41,42^ In SGZ, GFAP^+^/Sox2^+^ cells were classified as type-1 stem cells and GFAP^−^/Sox2^+^ cells as type-2 stem cells. We observed that HFD males (*P* = 0.038) and females (*P* = 0.025) had higher type-1 stem cell density compared to NFD males and females respectively. Type-2 stem cell density in HFD and EmbHFD males and females were not different from NFD males and females, respectively (Fig. 5B and C). EmbHFD males had higher type-2 stem cell density compared to HFD males (*P* = 0.019). There was no effect of sex on type-1 and -2 stem cell densities.

NSC markers (*Notch1, Sox2* and *Pax6*) were used to complement our immunostaining results in the hippocampus.^43,44^ In males and females, the mRNA levels of *Notch1, Sox2* and *Pax6* in HFD and EmbHFD groups were not different from those in the NFD group (Fig. 5D and E). These data suggest NSCs are not in an active division process, and this is consistent with the absence of changes in type-2 cell density.

These results suggest that maternal HFD may increase the reservoir of stem cells in adult offspring in the SGZ of the DG, possibly leading to changes in the production of other cell populations. Therefore, we analysed the overall cell (DAPI^+^) densities in the hippocampus. HFD males had higher cell density in the GCL (*P* = 0.015) but a lower cell density in SGZ compared to NFD males (*P* = 0.031) and a lower density in hilus compared to EmbHFD males (*P* = 0.029) (Fig. 5F). HFD and EmbHFD females had a lower cell density in the GCL (*P* = 0.019, *P* = 0.009) but a higher cell density in the hilus in the HFD females compared to NFD females (*P* = 0.028) (Fig. 5G). EmbHFD females had a lower cell density in the GCL (*P* = 0.009) compared to NFD females. To determine which cell population was responsible for these density changes, we analysed neuronal, astrocytic and microglial cell densities.

### Maternal HFD altered neuronal differentiation and maintenance in offspring hippocampus

**Figure 6:**
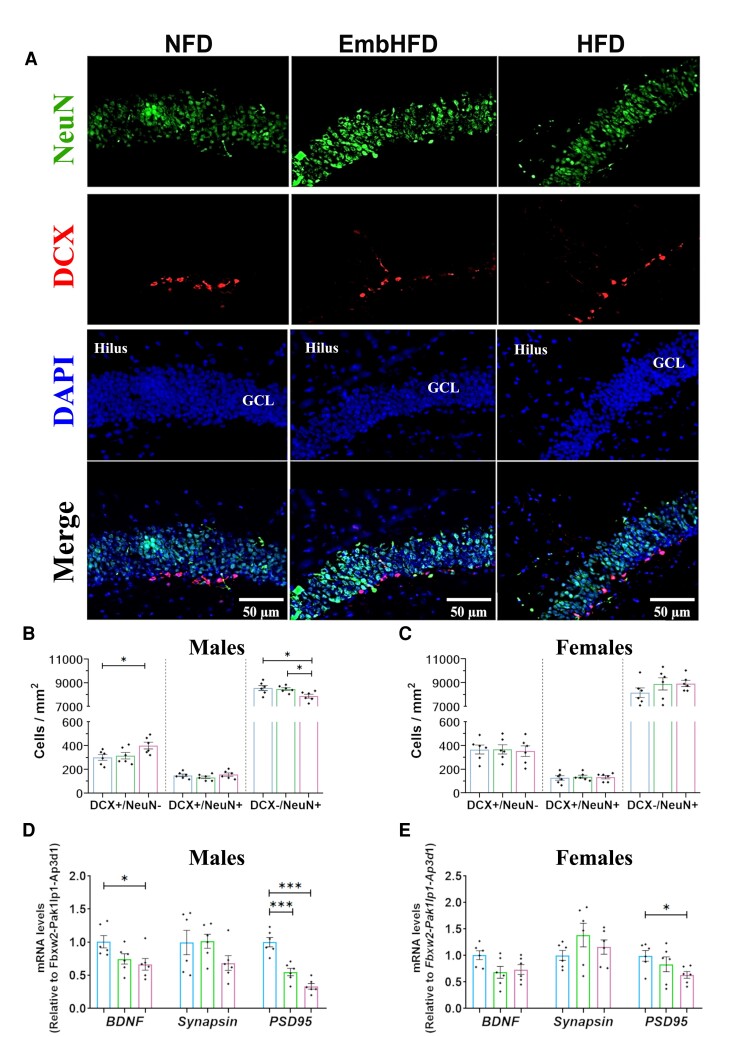
**Maternal HFD and EmbHFD increase newborn neurons but decrease mature neurons in the male adult offspring hippocampus. A.** Images of coronal DG sections analysed for DCX (red top row), NeuN (green second row), DAPI (blue third row), and merge (bottom row). **B.** In males, the density of newborn neurons (DCX+/NeuN–) and mature neurons (DCX–/NeuN+) in the HFD group was significantly different compared to the NFD group. EmbHFD males were significantly different from NFD males in the DCX–/NeuN+ cells. **C**. The density of cells in females did not change significantly between the diet groups, **D.** In HFD males the relative mRNA levels of *Bdnf*, and *Psd95*, were reduced when compared to the NFD males. EmbHFD males showed lower levels of *Psd95* than NFD males. **E.** In HFD females, the *Psd95* mRNA level was downregulated compared to the NFD females. NFD group (blue), EmbHFD group (green) and HFD group (red). Data were analysed by multilevel random effects regression and shown as dot plots with mean and SEM, (NFD, *n* = 6; EmbHFD, *n* = 6; HFD, *n* = 6, one animal per litter per sex), (**B, C, D**, and **E**). In the hippocampus, the mRNA levels of the selected markers were analysed by qPCR and normalized to *Fbxw2, Pak1lp1* and *Ap3d1*. Scale bars, 50 μm. *P < 0.05, ***P < 0.001.

Immature neurons express DCX which contributes to adult neurogenesis.^45,46^ In the adult hippocampus, NeuN is expressed by all post-mitotic cells and is used to distinguish different neuronal populations by double labelling with other markers such as DCX, calretinin or calbindin.^47^ Therefore, to investigate the extent of neuronal differentiation, neurons were double-stained with DCX and NeuN in the DG to estimate the number of three different cell types: newborn neurons (DCX^+^/NeuN^−^), immature neurons (DCX^+^/NeuN^+^), and mature neurons (DCX^−^/NeuN^+^) (Fig. 6A). In HFD males, newborn neuron density was increased (*P* = 0.033) and mature neuron density was decreased (*P* = 0.019) compared to NFD males while no differences were found in immature neuron density (Fig. 6B). The densities of newborn, immature and mature neurons were not changed in EmbHFD and HFD females (Figure 6C). EmbHFD males had a higher mature neuron (DCX^−^/NeuN^+^) density than HFD males (Supplementary Table 3). This indicates maternal HFD reduces neuronal differentiation in males, thereby reducing mature neurons possibly by a delay in this process, with cells accumulating at the newborn neuronal stage.

### Maternal HFD and EmbHFD decreased hippocampal plasticity markers in offspring

Neural plasticity defines the ability of the brain to adapt and respond to different challenges through cellular and molecular mechanisms of synapse formation, neurite growth and behavioural adaptation. Since maternal HFD modified the cellular density of neurons in adult offspring, we evaluated three markers involved in synaptogenesis, *PSD-95, Synapsin* and *BDNF*. In HFD males, *Bdnf* and *Psd95* expression was reduced (*P* = 0.022, and *P* = 0.00001, respectively) whereas *Synapsin* was unchanged compared to NFD males (Fig. 6D). In HFD females, *Psd95* was decreased (*P* = 0.037) but no changes in *Bdnf* and *Synapsin* expression were observed compared to NFD females. EmbHFD males showed decreased *Psd95* (*P* = 0.00045) (Fig. 6E). Moreover, increased *Synapsin* and *Psd95* expression (*P* = 0.059, and *P* = 0.110 respectively) were found in EmbHFD females and *Psd95* in HFD females compared to EmbHFD and HFD males, respectively (Supplementary Table 3). Maternal HFD and EmbHFD, therefore, decrease hippocampal plasticity markers in offspring, suggesting reduced hippocampal synapse density.

### Maternal HFD and EmbHFD increased hippocampal astrocytic density in offspring

**Figure 7:**
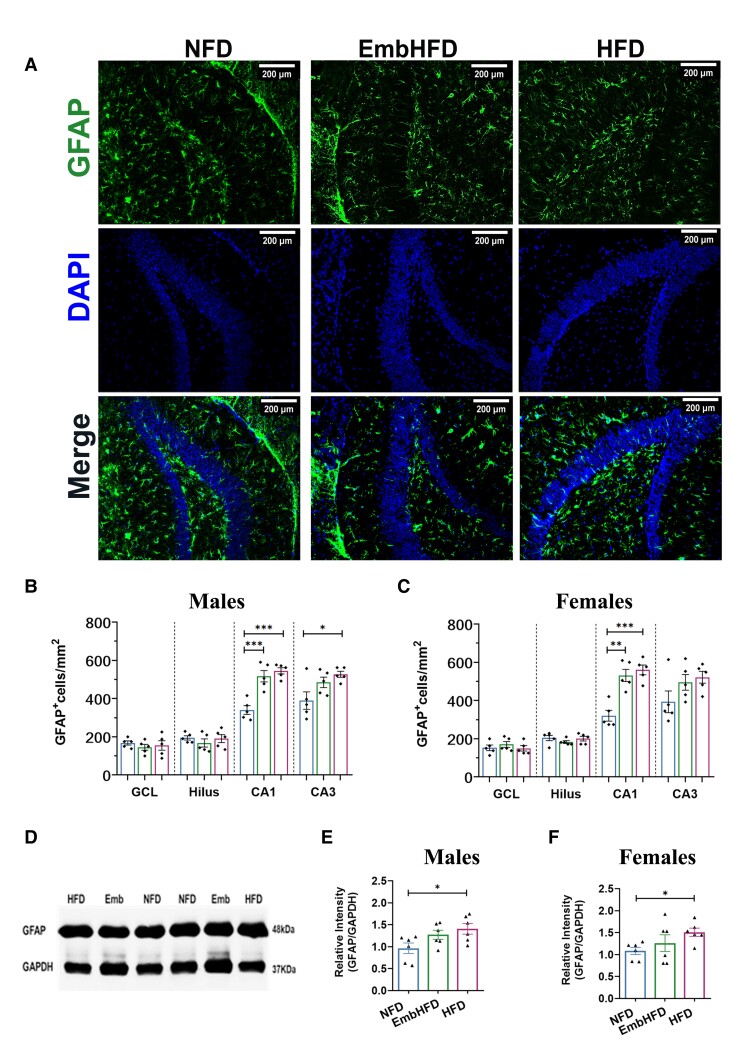
**GFAP astrocytic density is increased by maternal HFD and EmbHFD in the male and female adult offspring hippocampus**. **A**. Coronal sections with DAPI staining (blue, top row); Positive Astrocytes for GFAP (green, middle row) merged channels for GFAP+/DAPI+ cells (bottom row) in the hippocampus of mice from the different maternal diet groups. **B**. In males, the density of GFAP+ cells in the CA1 region was increased in HFD and EmbHFD compared to the NFD males, and the CA3 region in the HFD compared to the NFD males. **C**. In females, the number of GFAP+ cells was increased in HFD and EmbHFD in the CA1 region compared to the NFD females. **D.** Representative Western Blot used for the analysis of GFAP detected as a band at 48 kDa (top band) and GAPDH (bottom band) at 37 kDa. (*See*Supplementary Fig. 3*for uncropped blots*). **E.** In males, the protein expression levels of GFAP were higher in the HFD group compared to the NFD group. **F.** In females, the HFD group showed higher protein expression levels of GFAP compared to the NFD group. NFD group (blue), EmbHFD group (green) and HFD group (red) at 26 weeks of age. Data were analysed by multilevel random effects regression and shown as dot plots with mean and SEM, (NFD, *n* = 6; EmbHFD, *n* = 6; HFD, *n* = 6), (**B, C, D** and **E**). Each animal belonged to a single litter. The scale bar represents 200 μm. *P < 0.05, **P < 0.01, ***P < 0.001, ****P ≤ 0.0001.

We next investigated whether maternal diets altered the population of astrocytes in adult offspring. Despite the non-uniform distribution of GFAP-positive astrocytes in the brain, we decided to use GFAP since astrocytes in the hippocampus are highly GFAP-positive (Fig. 7A).^48,49^ Microscopy analysis revealed that GFAP^+^ cell density was increased in HFD males CA1 and CA3 (*P* = 0.00024, and *P* = 0.021, respectively) and in females CA1 (*P* = 0.00035) compared to NFD (Fig. 7B and C). GFAP^+^ cell density was also increased in EmbHFD CA1 for both males and females compared to NFD (*P* = 0.00093 and *P* = 0.004). No differences were found in the GCL and Hilus of the DG, and no sex differences were found. Western blot analysis on hippocampus whole cell lysates showed increased GFAP protein expression in HFD males and females (*P* = 0.025 and *P* = 0.045, respectively) but not in EmbHFD males and females (Fig. 7D–F).

### Maternal HFD and EmbHFD increased hippocampal microglial density in offspring

**Figure 8:**
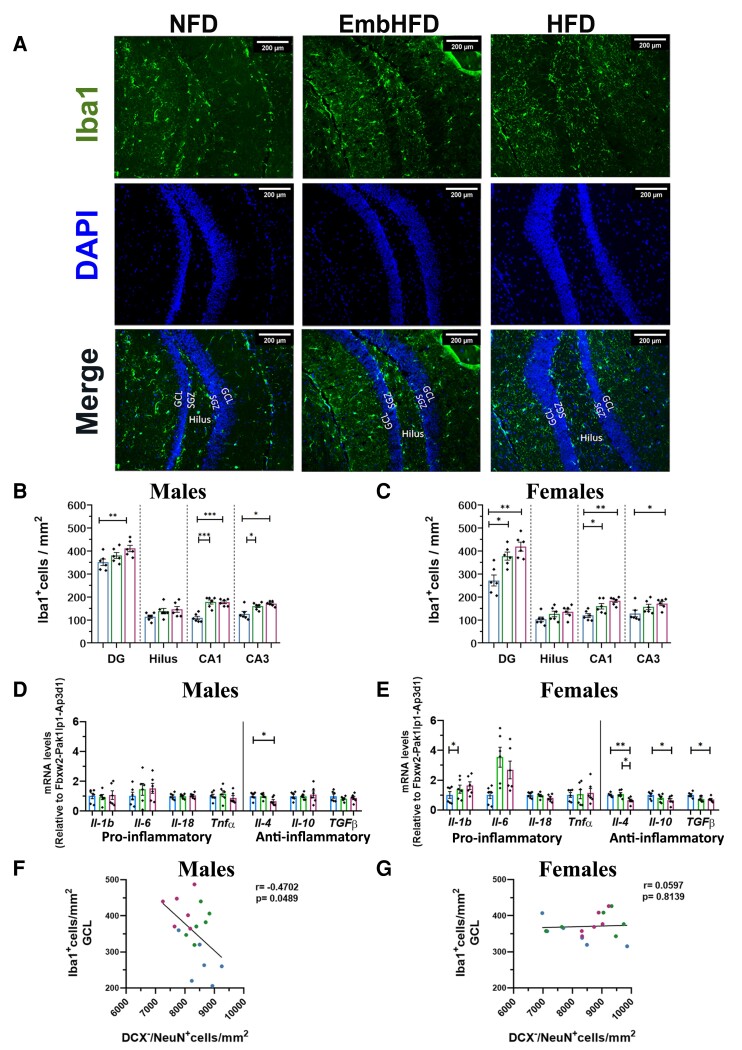
**Maternal HFD and EmbHFD increase hippocampal microglial density in the male and female adult offspring**. **A.** Coronal hippocampal sections stained with DAPI (blue, top row); Iba1 as a marker for microglia cells (green middle row), and double staining Iba1^+^/DAPI^+^ (bottom row). **B.** In males, there were differences in the density of Iba1^+^ cells in the DG, CA1 and CA3 in the HFD group, and CA1 and CA3 in the EmbHFD group compared to the NFD group. **C.** In females, there were differences in the density of Iba1^+^ cells in the DG, CA1 and CA3 in the HFD group, and DG and CA1 in the EmbHFD group compared to the NFD group. **D.** In males, the mRNA levels of the *Il-4* gene were significantly decreased in the HFD group compared to the NFD group. **E.** In females, the mRNA levels of the *Il-b* gene were significantly increased in the EmbHFD group compared to the NFD group. The mRNA levels of *Il-4*, *Il-10* and *Tgfβ* genes were significantly decreased in the HFD compared to the NFD group, and EmbHFD females were significantly different from HFD females in the mRNA levels of the *Il-4* gene. **F.** There is a negative correlation between the number of mature neurons (DCX–/NeuN+) and the number of microglia cells in males, but not females (**G**). Data were analysed by multilevel random effects regression and shown as dot plots with mean and SEM (**B, C, D** and **E**). Data were analysed by Pearson’s correlation between a number of Iba1^+^ cells and the number of DCX^−^/NeuN^+^ cells in the GCL (F and G) and shown as dot plot correlation analysis. Each animal belonged to a single litter NFD group (blue), EmbHFD group (green) and HFD group (red) at 26 weeks of age. (NFD, *n* = 6; EmbHFD, *n* = 6; HFD, *n* = 6). The scale bar represents 100μm. The mRNA levels of the selected markers were analysed by qPCR and normalized to *Fbxw2, Pak1lp1* and *Ap3d1.* The scale bar represents 200 μm. *P < 0.05, **P < 0.01, ***P < 0.001, ****P ≤ 0.0001.

In the adult brain, microglia cells continually examine the local environment through their processes.^50^ Maternal diet influences microglial function; for instance, HFD increases the number of microglia and apoptotic cells in the DG, indicating a detrimental effect on the survival of the new cells.^51^ We analysed the density of positive Iba1^+^ cells in the DG (GCL and SGZ), hilus, CA1 and CA3 of the hippocampus (Fig. 8A). HFD males and females had an increased density of Iba1^+^ cells in the DG (*P* = 0.002 and *P* = 0.000025), CA1 (*P* = 0.00024 and *P* = 0.001) and CA3 (*P* = 0.002 and *P* = 0.026) compared to the NFD males and females, respectively (Fig. 8B and C). EmbHFD males had increased Iba1^+^ cell density in the CA1 (*P* = 0.00026) and CA3 (*P* = 0.012) compared to NFD males, whereas EmbHFD females showed increased Iba1^+^ cell density in the DG (*P* = 0.000146) and CA1 (*P* = 0.039) compared to NFD females (Fig. 8B and C). Also, NFD males displayed higher Iba1^+^ cell density than females in the DG (Supplementary Table 3).

### Maternal HFD did not cause hippocampal neuroinflammation in offspring

We analysed the expression of genes involved in immune homeostasis including pro-inflammatory cytokines (interleukins *Il-1b, Il-6*, *Il-18*, *Tnfα*) and anti-inflammatory cytokines (interleukins *Il-4*, *Il-10*, *Tgfβ*) in the hippocampus. *Il-4* expression was decreased in HFD males (*P* = 0.027) (Fig. 8D) while *Il-1b* expression was increased in EmbHFD females (*P* = 0.017) (Fig. 8E). Anti-inflammatory cytokine genes *Il-4* (*P* = 0.004), *Il-10* (*P* = 0.027) and *Tgfβ* (*P* = 0.010) were decreased in HFD females but not in EmbHFD females (Fig. 8E). Thus, we show a decrease in the expression of anti-inflammatory mediators in the hippocampus in the HFD females, without a significant increase in pro-inflammatory mediators, suggesting that maternal HFD does not cause hippocampal neuroinflammation in adult offspring.

### Mature neuron density is inversely correlated with microglial cell density in male offspring hippocampus

Microglia are crucial elements in the adult hippocampal neurogenic niche since they regulate neurogenesis through phagocytosis. Most of the new cells in the adult DG cell debris are cleared by the microglia.^52^ Considering our findings on newborn and mature neuron densities in HFD males, we conducted a correlation analysis between neurogenesis and microglia. There were no significant correlations in the GCL between microglial cells and newborn cells or immature neurons in males or females. However, there was a significant negative correlation in the GCL between microglial (Iba1^+^) cells and mature (DCX^−^/NeuN^+^) neurons in males but not females across diet groups (Fig. 8F and G). This suggests that the mature neuron population may be phagocytosed by microglial cells, as mature neurons are decreased when microglia are increased in HFD males.

## Discussion

The nutritional status during pregnancy and lactation is essential for proper programming and development of the neural system that regulates behaviour and energy balance in the offspring. This implies that nutritional changes in the mother during pregnancy lead to physiological and behavioural alterations in the offspring’s life.^53,54^ This study showed that maternal HFD during the preimplantation period or during pregnancy and lactation significantly impacts adult offspring’s metabolic parameters and neuronal/glial markers in the absence of maternal obesity. These findings provide strong support for the DOHaD hypothesis and the vulnerability of the prenatal period, including the preimplantation phase of embryo development, to environmental conditions.^6,8,10^ We found that maternal HFD and EmbHFD in the absence of obesity have adverse effects on hippocampal neurogenesis and energy metabolism, indicating that transient poor nutrition in healthy mothers may contribute to adverse health in their offspring.

We found that maternal HFD did not alter the offspring's body weight, similar to other models where HFD was given just before or after mating.^55,56^ At week 26, an increase in diurnal activity was observed in HFD offspring females. Although the biological mechanism underlying this increase is unclear, the results suggest that the offspring of HFD dams (gestation/lactation) were more active during their resting period (daytime); this may be comparable to the hyperactivity observed in offspring of obese mothers in humans.^57^ Also, male and female HFD offspring developed elevated SBP. The nutritional environment to which mothers are exposed during pregnancy has previously been shown to influence CVD development in offspring.^58,59^ Additionally, in obese maternal models, prolonged maternal HFD before pregnancy leads to raised SBP in the offspring.^60,61^ The novelty of our model is that here we have shown in mice that exposure to a maternal HFD during gestation and lactation, in the absence of obesity, is associated with elevated SBP in adult offspring.

Mouse models of diet-induced obesity have shown increased SBP and leptin levels.^62^ Leptin is thought to contribute to the pathogenesis of hypertension by stimulating vascular smooth muscle dysfunction and vascular inflammation.^62,63^ In addition, it has been shown that leptin can utilize the glutamate pathway to control blood pressure.^64^ In the hippocampus, glutamate is essential for long-term potentiation and thus for memory formation. Similarly, it has been described that blood pressure affects not only hippocampal glutamate status but also memory function by affecting hippocampal integrity.^65^ Here we have seen that leptin receptors in the hippocampus are upregulated, and although we did not assess leptin in this study, it could be suggested that leptin might be related to elevated SBP in offspring.

Additionally, we found that maternal HFD affected the volume of oxygen consumed and carbon dioxide produced during the day and night in males from HFD-fed mothers during pregnancy and lactation. Lower oxygen consumption can increase blood pressure, which is necessary to increase oxygen delivery to different body tissues.^66,67^ Interestingly, HFD males and females showed higher RQ values during the day and night, and EmbHFD males and females showed increased RQ values in parts of the day but not during the night. RQ indicates which macronutrients are metabolized, with a value of 0.7 indicating that lipids are metabolized, 0.8 for protein and 1.0 for carbohydrates.^68^ The data suggest that maternal HFD could lead the offspring to rely primarily on carbohydrates and a reduced ability to use lipids as an energy source in adult offspring, thus these animals may have a metabolic inflexibility, as we expected maternal HFD to lead to decreased RQ in the offspring. Multiple studies have shown that regulation of EE is an effective way to prevent obesity,^69,70^ in concordance with our observations, that our HFD males had reduced EE. HFD has been reported to reduce EE due to a positive energy balance relative to energy intake.^71,72^ Choi *et al*. described that HFD-induced reduction in EE could be caused by the down-regulation of genes involved in fatty acid catabolism and oxidation of genes controlling energy transduction pathways in mitochondria.^72^ These studies support the idea that maternal HFD could affect the different metabolic parameters studied.

In the brain, glucose transporters facilitate glucose transport across the Blood Brain Barrier and glucose uptake into neurons.^36,73^ In the hippocampus, we observed decreased glucose transporters in HFD and EmbHFD, males and females, as shown previously for Slc2a1 in a rat model using a maternal 60% HFD during gestation and lactation.^74^ Reducing these transporters in the hippocampus can modify behaviour and, specifically, learning.^75,76^ Slc2a3, mainly found in neurons, is the major glucose transporter in the cortex and hippocampus.^77^ Heterozygous null *Slc2A3* mice show normal body development and brain size but display cognitive abnormalities.^78,79^ Slc2a4 and Slc2a8 are expressed in neurons and are insulin-sensitive glucose transporters located in selective areas of the brain, such as the hippocampus, hypothalamus, and cortex, and Slc2a5 is expressed in microglia.^78,80^*Slc2A8*−/− mice have been associated with neurogenesis and hyperactivity, suggesting that Slc2A8 could work as a neurogenic regulator.^40,80,81^ Here, we show an increased number of newborn neurons and a decreased *Slc2A8* expression in the male HFD offspring. The reduction in glucose transporters’ mRNA levels in the hippocampus observed in our study may reflect an impairment in neuronal–glial interactions for nutrient transport.^73^ However, to date, no model correlates reduced expression of glucose transporters caused by a maternal HFD and cognitive deficits or memory impairment in adulthood.

The main function of adiponectin, secreted exclusively by adipose tissue, is to increase insulin sensitivity.^37,82^ Adiponectin binds to two membrane receptors, AdipoR1 and AdipoR2, stimulating *Slc2A4* transcription.^83^ In the present study, the mRNA levels of *AdipoR1, AdipoR2* and *Slc2A4* decreased in the hippocampus of adult HFD male offspring. Reduced expression of *AdipoR1* and *AdipoR2* induced by HFD and altered neurogenesis in the hippocampus suggests that AdipoR1 signalling might be neuroprotective against metabolic insults.^38^

In the adult DG, we observed an increase in radial glial cells in HFD males and females and an increase in newborn neurons and a decrease in mature neurons and synaptic markers in HFD males. This suggests an imbalance of proliferation and cell death at different stages of the neurogenesis process. Although the molecular mechanisms underlying these changes in neurogenesis have not yet been elucidated. The Notch/Hes signalling pathway is highly expressed in the adult hippocampus, as it regulates the reserve, maintenance, and proliferation of neural stem cells. It has been reported that in the offspring of obese mothers after HFD consumption this pathway could delay or inhibit neurogenesis, suggesting the impact that maternal HFD has on the offspring.^21,84^ We observed an increased density of GFAP^+^ astrocytes and Iba1^+^ microglia in the hippocampus in the HFD and EmbHFD groups. Both astrocytes and microglia are involved in neuroinflammation. However, the analysis of inflammatory markers did not show significant induction of pro-inflammatory cytokines in the offspring of HFD-fed dams at 26 weeks of age (EmbHFD or HFD males or females). Astrocytes and microglia are also essential in synaptogenesis, with astrocytes interacting with presynaptic and postsynaptic neurons, forming the tripartite synapse^85^ and microglia pruning neurons with defective synapses.^86^ We showed a significant negative correlation between microglia and mature neurons, supporting the idea that the decrease in neurogenesis may result from a disruption in microglial surveillance capacity. Dias *et al.*, described that the offspring of obese mothers fed HFD showed neuroinflammation and increased BDNF expression in the hippocampus.^87^ We observed that BDNF and PSD95 expression was reduced at the hippocampal level in offspring exposed to maternal HFD (preimplantation or gestation/lactation). This difference could be due to the absence of obesity in the mothers in our study and the time of exposure of the offspring to the HFD. Low levels of BDNF in the hippocampus decrease spatial learning, synaptic and cognitive function, and PSD95 plays a critical role in the organization of postsynaptic spatial learning and visual cortical plasticity.^88,89^ Consistent with our findings, recent research has observed reduced BDNF levels in the offspring of obese HFD-fed mothers, suggesting that these changes may be due to a decrease in Serotonin (5HT1A), and with a possible impact on hippocampal neurogenesis.^20^ These changes in BNDF and PSD95 could suggest an altered synaptic function in the hippocampus and possibly spatial learning, synaptic function, and cognitive function.

Our findings indicate that exposure to maternal HFD affects hippocampal neurogenesis, induces the development of metabolic disorders, especially hypertension, and changes metabolic activity, such as EE, in the adult offspring. Moreover, the findings from the EmbHFD treatment restricted to the preimplantation period indicate generally a milder phenotype than HFD but a significant impact is still evident on aspects of energy metabolism and hippocampal cell density and gene expression. These results collectively provide further evidence for the importance of an adequate maternal diet from before conception as a healthy strategy to decrease susceptibility to developing neuronal and metabolic-related dysfunction in adulthood.

## Supplementary Material

fcad093_Supplementary_Data

## Data Availability

All data reported in this manuscript are stored at Southampton General Hospital, Faculty of Medicine and are available from the corresponding author upon reasonable request.

## Abbreviations

AUC =: area under the curve
BCA =: bicinchoninic acid
BDNF =: Brain-derived neurotrophic factor
BW =: Body weight
CA1 =: Cornu ammonis 1
CA3 =: Cornu ammonis 3
CVD =: Cardiovascular disease
DAPI =: 4,6-diamidino-2-phenylindole
DCX =: doublecortin
DG =: dentate gyrus
DOHaD =: developmental origins of health and disease
EE =: energy expenditure
EmbHFD =: embryonic high-fat diet
GAPDH =: Glyceraldehyde 3-phosphate dehydrogenase
GCL =: granule cell layer
GFAP =: glial fibrillary acidic protein
GTT =: glucose tolerance test
HFD =: high-fat diet
Iba1 =: ionized calcium binding adaptor molecule 1
IOD =: index of obesity degree
NCDs =: non-communicable diseases
NeuN =: neuronal nuclei
NIBP =: non-invasive blood pressure monitor
NFD =: normal fat diet
NSC =: Neural stem cells
PBST =: phosphate-buffered saline with Triton X-100
PVDF =: Polyvinylidene difluoride
RIPA buffer =: radioimmunoprecipitation assay buffer
RQ =: respiratory quotient
SBP =: systolic blood pressure
SDS =: special diet services
SGZ =: subgranular zone
Sox2 =: SRY-box transcription factor 2
