# Supplementary material for: Preimplantation or gestation/lactation high-fat diet alters adult offspring metabolism and neurogenesis
Source: Brain Commun. 2023 Mar 29;5(2):fcad093. doi: 10.1093/braincomms/fcad093 (PMC10077335; doi:10.1093/braincomms/fcad093)
Supplement: fcad093_Supplementary_Data [file fcad093_supplementary_data.docx]

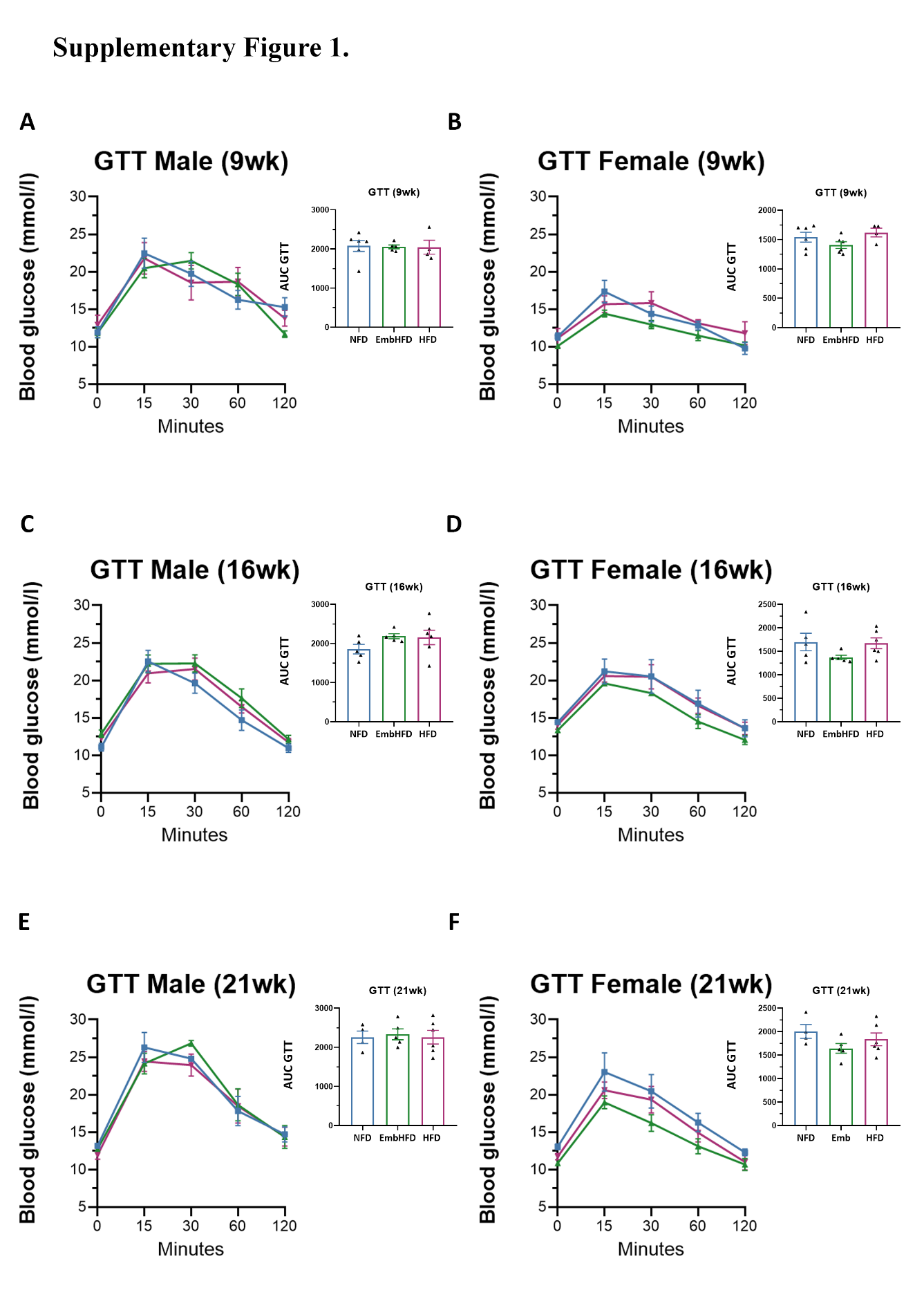


**Supplementary Figure 1. Effect of maternal HFD and EmbHFD on glucose tolerance test. a).** GTT in males at 9 weeks of age. **b)**. GTT in females at 9 weeks of age. **c).** GTT in males at 16 weeks of age. **d).** GTT in females at 16 weeks of age. **e)**. GTT in males at 21 weeks of age. **f).** GTT in females at 21 weeks of age. NFD group in blue, EmbHFD group in green and HFD group in red. Data are expressed as mean ± SEM. n=6-8 in each group. No significant differences in any of the data presented in this figure.


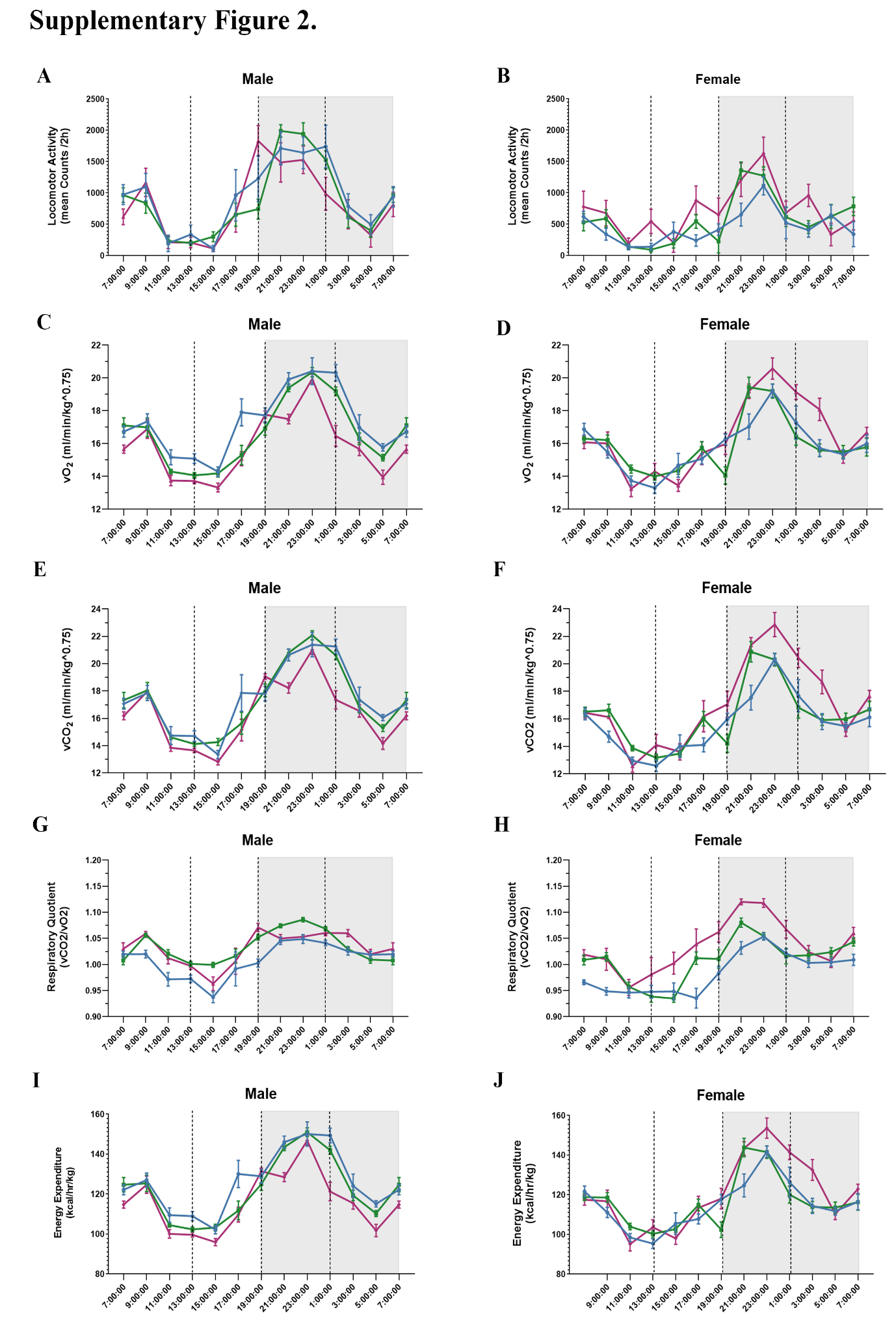


**Supplementary Figure 2.** **Effect of maternal HFD and EmbHFD on indirect calorimetry in the adult offspring. a).** Male locomotor activity over a 24 h period. **b).** Female locomotor activity over a 24 h period. **c).** Male vO_2_ consumption over a 24 h period **d).** Female vO_2_ consumption over a 24 h period. **e).** Male vCO_2_ consumption over a 24 h period. **f).** Female vCO_2_ consumption over a 24 h period. **g).** Male RQ over a 24 h period. **h).** Female RQ over a 24 h period. **i).** Male energy expenditure over a 24 h period. **j).** Female energy expenditure over a 24 h period. The shaded areas demarcate the dark phases (from 19:00 to 7:00). NFD group (blue), Emb group (green) and HFD group (red) at 26 weeks age. All points represent means ± SEM. n=4-6 in each group.

**
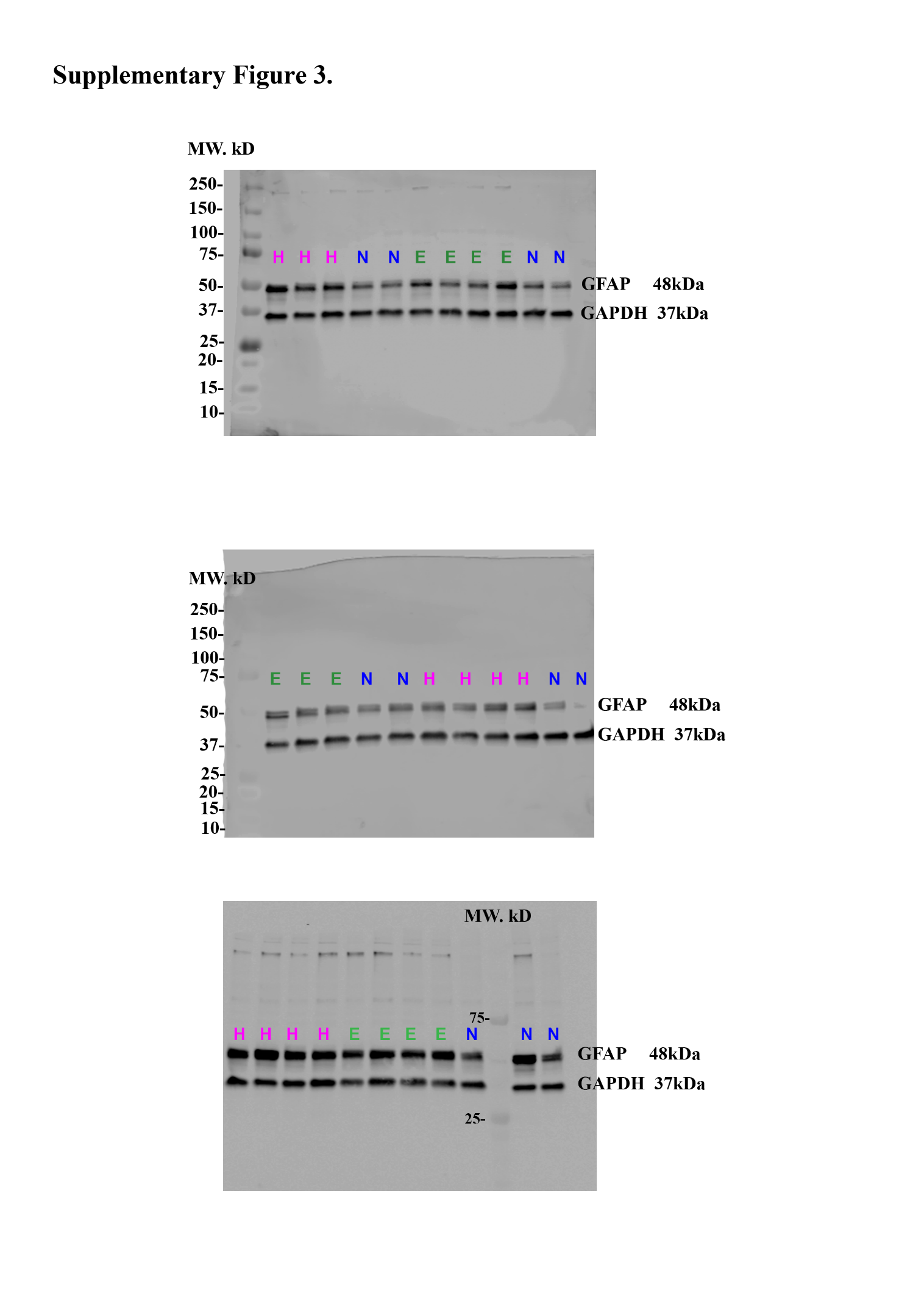
**

**Supplementary Figure 3. Extended western blots for the data shown in Figure 7.** N = NFD group, E = EmbHFD group, and H = HFD group.

**Su****pplementary Table 1.** Energy composition and fat breakdown of the normal fat diet and the high-fat diet.

*AFE=Atwater((CO%/100) *9000)+((CP%/100) *4000)+((NFE5/100) *4000)/239.23.

**Supplementary Table 2.** List of primer sequences used for qPCR. Sequences for primers for *B3-Tub*, *Notch-1, Pax6*, *Fbxw2, Pak1lp1, Pgk1, Tbp* and *Ap3d1* are property of PrimerDesign, UK.

| **Gene** | **Gene Name** | **Accession No.** | **Primer Sequence** | |
| --- | --- | --- | --- | --- |
| ***AdipoR1*** | Adiponectin receptor 1 | NM_001306069 | F | AGTGCATGGTGGGTACAACA |
|  |  |  | R | AGCACCGGCAGACAAGAG |
| ***AdipoR2*** | Adiponectin receptor 2 | NM_001355692 | F | GGTGGCAGCCTTCAGGAACCC |
|  |  |  | R | AGAGCAGGAGTGTTCGTGGGCT |
| ***Bdnf*** | Brain derived neurotrophic factor | NM_001048139.1 | F | TCATACTTCGGTTGCATGAAGG |
|  |  |  | R | AGACCTCTCGAACCTGCCC |
| ***Slc2A1*** | Solute carrier family 2 (facilitated glucose transporter), member 1 | NM_011400.3 | F | AGAAGAGGGTCGGCAGATGA |
|  |  |  | R | AAGTTTGAGGTCCAGTTAGA |
| ***Slc2A3*** | Solute carrier family 2 (facilitated glucose transporter), member 3 | NM_011401.4 | F | GCCCCGAGAGTCCAAGGTTC |
|  |  |  | R | ACCAAGATAGCCACAATACA |
| ***Slc2A4*** | Solute carrier family 2 (facilitated glucose transporter), member 4 | NM_001359114 | F | GTTGGTCTCGGTGCTCTTAGT |
|  |  |  | R | TGCCACAATGAACCAGGGAA |
| ***Slc2A5*** | Solute carrier family 2 (facilitated glucose transporter), member 5 | NM_019741.3 | F | TCTTTGTGGTAGAGCTTTGGG |
|  |  |  | R | GACAATGACACAGACAATGCTG |
| ***Slc2A8*** | Solute carrier family 2, (facilitated glucose transporter), member 8 | NM_019488.5 | F | CAGCTGATGGTTGTCACTGG |
|  |  |  | R | CCAGCGCCACTCTAGGAC |
| ***Ifg1R*** | Insulin-like growth factor I receptor | NM_010513.2 | F | CACTTGCATGACGTCTCTCC |
|  |  |  | R | GAGAATTTCCTTCACAATTCCATC |
| ***Il-1b*** | Interleukin 1 beta | NM_008361.4 | F | GAAGAGCCCATCCTCTGTGA |
|  |  |  | R | TTCATCTCGGAGCCTGTAGTG |
| ***Il-4*** | Interleukin 4 | NM_021283.2 | F | CCTGCTCTTCTTTCTCGAATGT |
|  |  |  | R | TTTCAGTGATGTGGACTTGGAC |
| ***Il-6*** | Interleukin 6 | NM_001314054.1 | F | CTCTGCAAGAGACTTCCATCC |
|  |  |  | R | TGAAGTCTCCTCTCCGGACT |
| ***Il-10*** | Interleukin 10 | NM_010548.2 | F | AGGCGCTGTCATCGATTTCTC |
|  |  |  | R | TGCTCCACTGCCTTGCTCTTA |
| ***Il-18*** | Interleukin 18 | NM_001357221.1 | F | ACGTGTTCCAGGACACAACA |
|  |  |  | R | CAAACCCTCCCCACCTAACT |
| ***InsR*** | Insulin receptor | NM_001330056 | F | TCTTTCTTCAGGAAGCTACATCTG |
|  |  |  | R | TGTCCAAGGCATAAAAAGAATAGTT |
| ***ObRa*** | Leptin receptor a | NM_008493.3 | F | GAAGTCTCTCATGACCACTACAGATGA |
|  |  |  | R | TTGTTTCCCTCCATCAAAATGTAA |
| ***ObRb*** | Leptin receptor b | NM_008493.3 | F | GCATGCAGAATCAGTGATATTTGG |
|  |  |  | R | CAAGCTGTATCGACACTGATTTCTTC |
| ***Psd95*** | Discs large MAGUK scaffold protein 4 | NM_001109752.1 | F | GTACCTAAAGGTGGCCAAGC |
|  |  |  | R | GAGAGAGCTCGTTGTTGATAAGCT |
| ***Sox2*** | SRY-box transcription factor 2 | NM_011443.4 | F | TGGGCTCTGTGGTCAAGTC |
|  |  |  | R | TGATCATGTCCCGGAGGT |
| ***Synapsin*** | Synapsin I | NM_001110780.1 | F | CAGCACAACATACCCTGTGG |
|  |  |  | R | GGTCTTCCAGTTACCCGACA |
| ***Tnf-α*** | Tumor necrosis factor | NM_001278601.1 | F | TGCCTATGTCTCAGCCTCTTC |
|  |  |  | R | GAGGCCATTTGGGAACTTCT |
| ***Tgf-β*** | Transforming growth factor, beta 1 | NM_011577.2 | F | TGCTTCAGCTCCACAGAGAA |
|  |  |  | R | TACTGTGTGTCCAGGCTCCA |

**Supplementary table 3.** Sex differences in the different parameters evaluated. Significant p values (p<0.05) in bold.

|  |  |  | **NFD** | | | | | **EmbHFD** | | | | | **HFD** | | | | |
| --- | --- | --- | --- | --- | --- | --- | --- | --- | --- | --- | --- | --- | --- | --- | --- | --- | --- |
|  |  |  | **Males** | | **Females** | | ***p value*** | **Males** | | **Females** | | ***p value*** | **Males** | | **Females** | | ***p value*** |
|  |  |  | **Mean** | **SD** | **Mean** | **SD** |  | **Mean** | **SD** | **Mean** | **SD** |  | **Mean** | **SD** | **Mean** | **SD** |  |
| **Activity** | Day | 1st part | 3897.0 | 841.2 | 1712.0 | 391.4 | **0.0015** | 3319.0 | 717.4 | 2089.0 | 558.0 | 0.0670 | 3589.0 | 845.9 | 3068.0 | 862.0 | 0.8890 |
|  |  | 2nd part | 3720.0 | 1285.0 | 1796.0 | 534.1 | **0.0490** | 2860.0 | 779.9 | 1798.0 | 562.4 | 0.3620 | 3626.0 | 1041.0 | 3362.0 | 888.5 | 0.9980 |
|  | Night | 1st part | 33057.0 | 9101.0 | 11261.0 | 4234.0 | **0.0021** | 27132.0 | 5184.0 | 11786.0 | 4312.0 | **0.0089** | 31722.0 | 7305.0 | 16676.0 | 5176.0 | **0.0340** |
|  |  | 2nd part | 5255.0 | 1042.0 | 2963.0 | 735.8 | **0.0450** | 4533.0 | 903.2 | 3557.0 | 743.6 | 0.5750 | 3784.0 | 1199.0 | 3825.0 | 740.1 | 0.9980 |
| **vO2** | Day | 1st part | 96.9 | 2.0 | 88.6 | 1.7 | **0.0002** | 93.8 | 2.6 | 91.6 | 1.4 | 0.5610 | 90.7 | 2.2 | 88.9 | 2.6 | 0.8190 |
|  |  | 2nd part | 97.2 | 2.7 | 89.1 | 2.4 | **0.0007** | 90.0 | 2.3 | 88.3 | 1.7 | 0.8290 | 88.3 | 1.6 | 88.0 | 2.5 | 0.9980 |
|  | Night | 1st part | 420.4 | 12.3 | 371.5 | 19.3 | **0.0006** | 401.0 | 11.6 | 346.5 | 13.6 | **0.0001** | 380.9 | 13.5 | 391.1 | 14.3 | 0.8790 |
|  |  | 2nd part | 102.6 | 2.6 | 95.4 | 2.8 | **0.0030** | 99.2 | 2.1 | 94.4 | 2.2 | **0.0430** | 91.4 | 2.4 | 102.3 | 2.4 | **0.0004** |
| **vCO2** | Day | 1st part | 97.0 | 2.6 | 84.3 | 1.9 | **0.0001** | 96.8 | 2.7 | 90.7 | 1.8 | **0.0080** | 93.5 | 1.1 | 88.0 | 1.9 | **0.0180** |
|  |  | 2nd part | 95.0 | 4.1 | 84.8 | 2.9 | **0.0020** | 92.0 | 3.0 | 86.4 | 2.1 | 0.0980 | 88.7 | 2.4 | 90.8 | 3.9 | 0.9450 |
|  | Night | 1st part | 432.1 | 13.9 | 374.4 | 22.5 | **0.0011** | 429.6 | 13.5 | 355.6 | 18.3 | **0.0002** | 404.3 | 14.2 | 420.2 | 21.5 | 0.7320 |
|  |  | 2nd part | 105.2 | 2.9 | 96.4 | 3.2 | **0.0020** | 102.2 | 2.5 | 97.3 | 2.6 | 0.0940 | 95.0 | 2.6 | 105.8 | 3.2 | **0.0001** |
| **EE** | Day | 1st part | 704.2 | 15.9 | 686.8 | 18.8 | **0.0012** | 664.0 | 16.0 | 636.3 | 12.6 | 0.5830 | 664.3 | 10.9 | 645.0 | 20.0 | 0.5850 |
|  |  | 2nd part | 702.4 | 21.8 | 657.5 | 18.2 | **0.0010** | 642.5 | 12.5 | 639.8 | 17.8 | 0.5740 | 638.1 | 12.9 | 644.7 | 20.2 | 0.9980 |
|  | Night | 1st part | 3076.0 | 91.6 | 2965.0 | 87.2 | **0.0008** | 2810.0 | 99.2 | 2705.0 | 145.3 | **0.0020** | 2534.0 | 105.9 | 2894.0 | 116.0 | 0.8410 |
|  |  | 2nd part | 750.1 | 19.4 | 726.1 | 15.7 | **0.0031** | 670.5 | 17.6 | 695.1 | 21.1 | **0.0490** | 691.3 | 16.2 | 750.0 | 19.0 | **0.0001** |
| **RQ** | Day | 1st part | 6.0 | 0.0 | 6.2 | 0.0 | 0.0520 | 6.2 | 0.0 | 5.7 | 0.0 | 0.0670 | 5.9 | 0.0 | 5.9 | 0.1 | 0.0610 |
|  |  | 2nd part | 5.8 | 0.1 | 6.1 | 0.1 | 0.2630 | 6.0 | 0.1 | 5.7 | 0.1 | 0.0640 | 5.8 | 0.1 | 6.1 | 0.1 | 0.3780 |
|  | Night | 1st part | 22.5 | 0.2 | 23.4 | 0.2 | 0.3880 | 23.4 | 0.2 | 22.1 | 0.3 | **0.0060** | 22.5 | 0.4 | 23.6 | 0.5 | 0.9320 |
|  |  | 2nd part | 6.2 | 0.0 | 6.2 | 0.0 | 0.0630 | 6.3 | 0.0 | 6.0 | 0.0 | 0.9980 | 6.1 | 0.0 | 6.2 | 0.1 | 0.9898 |
| **DCX-/NeuN+ (Cells/mm^2^)** | | | 8545.9 | 524.2 | 8082.5 | 1062.8 | 0.2660 | 8465.1 | 284.1 | 8892.4 | 1259.4 | 0.7650 | 7865.6 | 403.1 | 8922.2 | 643.3 | 0.2400 |
| **Psd95 (mRNA levels)** | | | 1.0 | 0.2 | 1.0 | 0.3 | 0.9610 | 0.5 | 0.1 | 0.8 | 0.3 | 0.2800 | 0.0 | 0.1 | 0.6 | 0.2 | 0.1600 |
| **Synapsin (mRNA levels)** | | | 1.0 | 0.5 | 1.0 | 0.3 | 0.9980 | 1.0 | 0.3 | 1.7 | 0.8 | **0.0390** | 0.7 | 0.3 | 1.2 | 0.3 | 0.0550 |
